# Combined lineage tracing and scRNA‐seq reveal the activation of Sox9^+^ cells in renal regeneration with PGE_2_ treatment

**DOI:** 10.1111/cpr.13679

**Published:** 2024-05-27

**Authors:** Shang Chen, Yue Liu, Xiaoniao Chen, Hongyan Tao, Yongjun Piao, Haoyan Huang, Zhibo Han, Zhong‐Chao Han, Xiang‐Mei Chen, Zongjin Li

**Affiliations:** ^1^ Nankai University School of Medicine Tianjin China; ^2^ The Key Laboratory of Bioactive Materials, Ministry of Education Nankai University Tianjin China; ^3^ Department of Ophthalmology The Third Medical Center of Chinese PLA General Hospital Beijing China; ^4^ National Key Laboratory of Kidney Diseases Chinese PLA General Hospital Beijing China; ^5^ MRC Molecular Hematology Unit, MRC Weatherall Institute of Molecular Medicine, John Radcliffe Hospital University of Oxford Oxford UK; ^6^ Academy of Medical Engineering and Translational Medicine Tianjin University Tianjin China; ^7^ Tianjin Key Laboratory of Engineering Technologies for Cell Pharmaceutical, National Engineering Research Center for Cell Products AmCellGene Co., Ltd. Tianjin China; ^8^ Tianjin Key Laboratory of Human Development and Reproductive Regulation, Tianjin Central Hospital of Gynecology Obstetrics Nankai University Affiliated Hospital of Obstetrics and Gynecology Tianjin China; ^9^ Henan Key Laboratory of Cardiac Remodeling and Transplantation Zhengzhou No. 7 People's Hospital Zhengzhou China

## Abstract

Uncovering mechanisms of endogenous regeneration and repair through resident stem cell activation will allow us to develop specific therapies for injuries and diseases by targeting resident stem cell lineages. Sox9^+^ stem cells have been reported to play an essential role in acute kidney injury (AKI). However, a complete view of the Sox9^+^ lineage was not well investigated to accurately elucidate the functional end state and the choice of cell fate during tissue repair after AKI. To identify the mechanisms of fate determination of Sox9^+^ stem cells, we set up an AKI model with prostaglandin E2 (PGE_2_) treatment in a Sox9 lineage tracing mouse model. Single‐cell RNA sequencing (scRNA‐seq) was performed to analyse the transcriptomic profile of the Sox9^+^ lineage. Our results revealed that PGE_2_ could activate renal Sox9^+^ cells and promote the differentiation of Sox9^+^ cells into renal proximal tubular epithelial cells and inhibit the development of fibrosis. Furthermore, single‐cell transcriptome analysis demonstrated that PGE_2_ could regulate the restoration of lipid metabolism homeostasis in proximal tubular epithelial cells by participating in communication with different cell types. Our results highlight the prospects for the activation of endogenous renal Sox9^+^ stem cells with PGE_2_ for the regenerative therapy of AKI.

## Graphical abstract

PGE_2_ activated renal Sox9^+^ cells and promoted the differentiation of Sox9^+^ cells into renal proximal tubular epithelial cells, further inhibiting the development of fibrosis. Single‐cell transcriptome analysis demonstrated that PGE_2_ could activate endogenous renal Sox9^+^ stem cells through the Pi3k‐Akt signalling pathway, promote the secretion of VEGFa, and maintain the metabolic homeostasis of kidneys via upregulation of Cpt2.

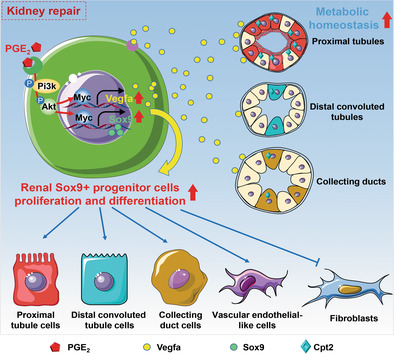

## 1 INTRODUCTION

The mammalian kidneys are a highly complex organ, consisting of more than 30 different types of cells.^1^ More than 10 types of potential stem or progenitor cells have been identified, and it is a challenge to unravel these interactions and establish lineage relationships during kidney homeostasis and injury repair. Genome‐wide transcriptome data obtained by single‐cell RNA sequencing (scRNA‐seq) analysis could be used to fairly map cell heterogeneity and independently restore cell identity, providing an a priori defined labelling strategy. The different cell types that descend from the same lineage can be identified accurately and precisely using scRNA‐seq.^2^ The combination of scRNA‐seq and lineage tracing will provide information on cell end fate and detailed cell phenotypes.^3^

Activating endogenous stem cells offers exciting potential for kidney regeneration after injury. Strategy through regulation of an instructive microenvironment is needed to recruit and stimulate resident renal progenitor cells or stem cells in vivo, to provide signals for native healing cascades, and to promote cell proliferation and differentiation of vulnerable proximal tubules for kidney repair after AKI.^4^, ^5^ Recent studies have shown that injury‐induced PGE_2_ secretion plays an important role in the regulation of endogenous stem cells for myocardial regeneration, intestinal regeneration and liver regeneration after injury.^6^, ^7^, ^8^, ^9^ Furthermore, Sox9 is upregulated in the early developing nephron and is located at the tips of the branching ureteral network where progenitors reside for the entire ureteral network.^10^ Much of the nonvascular epithelium in the kidney has been shown to be derived from a Sox9^+^ cell type.^11^ The proliferation of endogenous renal Sox9^+^ stem cells in the early phase of AKI promotes the repair of renal tubular epithelial cells, as detected in our previous study.^12^ However, the molecular mechanism of Sox9^+^ cell differentiation and heterogeneity in renal repair after IRI‐AKI has not yet been fully elucidated.

In this study, we effectively activated endogenous stem cells to repair the kidney using PGE_2_ and highlighted the potential of activated endogenous stem cells for application. Single‐cell sequencing analysis was used to examine the dynamics of kidney Sox9^+^ endogenous stem cell differentiation in Sox9 transgenic mice treated with PGE_2_. Furthermore, differentiation of the molecular mechanism of the activated endogenous stem cells and the dynamics of gene expression in the renal repair after AKI were investigated. Our objective was to identify the progeny subsets that regulate endogenous stem cell activation during kidney regeneration after AKI and uncover their functional states, differentiation and potential molecular regulators at single‐cell resolution.

## 2 MATERIALS AND METHODS

### 2.1 Sox9‐Cre^ERT2^ ; Rosa26^mTmG^ transgenic mice

C57BL/6 Sox9‐Cre^ERT2^ transgenic mice (#007676) and Rosa26^mTmG^ transgenic mice (#018829) were purchased from Jackson Laboratory; Sox9‐Cre^ERT2^ animals express a ligand‐dependent chimeric Cre recombinase (ERT2‐Cre) through the Sox9 promoter. Rosa26^mTmG^ mice expressed the fluorescent protein tdTomato (red) through the beta‐actin promoter. Cell labelling occurs only at the time of tamoxifen injection, EGFP was expressed in Sox9^+^ cell type‐specific cells, where the tdTomato and polyadenylation sequences were excised by Cre‐mediated recombination, but daughter cells retain the EGFP label, as genetic recombination cannot be reversed in the nucleus. Mice were injected with tamoxifen dissolved in corn oil intraperitoneally (100 mg/kg daily, mice were injected three times in the week before the experiment). Sox9‐Cre^ERT2^; Rosa26^mTmG^ transgenic mice were obtained by mating homozygotes from the two transgenic mice.^13^

### 2.2 AKI model and PGE_2_ therapy

The preparation of the PGE_2_ matrix was followed by the previous report.^12^, ^14^ In brief, PGE_2_ was chemically cross‐linked to collagen through the cross‐linker to obtain the collagen matrix that releases PGE_2_ (PGE_2_ matrix). The AKI model and PGE_2_ matrix delivery were performed as previously reported.^12^ In brief, mice (male) were anaesthesia by intraperitoneal injection with 2.5% avertin at a dosage of 240 mg/kg. The mice were clamped at the left renal pedicle for 40 min, followed by removal of the right kidney after injury. Reperfusion was visually confirmed before the delivery of the matrix. After 10 min of reperfusion, 75 μL of collagen matrix (control) or PGE_2_‐releasing matrix (PGE_2_ treatment, 2 μM) was injected into the renal capsule with an insulin syringe. The Sham group was treated with a sham operation and the wound was sutured. The PGE_2_ treatment group and collagen group (as control) were treated for 28 days after ischemia–reperfusion. Three Sox9‐Cre^ERT2^; Rosa26^mTmG^ transgenic mice in each group were utilized for the scRNA‐seq analysis. The treatment of the animals and the experimental procedures of the present study were in accordance with the guidelines of the Nankai University Animal Care and Use Committee (approval no. IRM‐DWLL‐2019121), which is in accordance with the Guidelines for Animal Care approved by the National Institutes of Health (NIH).

### 2.3 Flow cytometry and cell sorting

On Day 28 after PGE_2_ treatment, mice were sacrificed, kidney tissues were collected and then mechanically minced using a sterile scalpel (Feather), and three kidneys were dissociated using Multitissue Dissociation Kit 2 (Miltenyi Biotec) following the manufacturer's instructions. EGFP‐positive cells were sorted using a Multicolor Flow Cytometer (BD LSRFortessa X‐20). The viability of isolated cells was determined by trypan blue exclusion. Data were analysed using Flow Jo software (version X.0.7, Tree Star Inc.).

### 2.4 Analysis of renal functions

For the assessment of renal function, on Day 28 after injury, blood samples were harvested, and serum was collected for the assessment of blood urea nitrogen (BUN) and serum creatinine (sCr) using the Urea Nitrogen (BUN) Colorimetric Detection Kit (C013‐1, Nanjing Jiancheng Bioengineering Institute) and Creatinine Assay Kit (C011, Nanjing Jiancheng Bioengineering Institute).

### 2.5 Statistical analysis

Data were analysed with GraphPad Prism software (San Diego, CA). One‐way repeated‐measures ANOVA with Tukey post hoc tests were used. Differences were considered significant at a *p* value <0.05. Markers defining each cluster, as well as differential gene expression between different clusters, were calculated using a two‐sided Wilcoxon rank‐sum test that was implemented in Seurat.

## 3 RESULTS

### 3.1 Sox9^+^ cell activation after AKI

**FIGURE 1:**
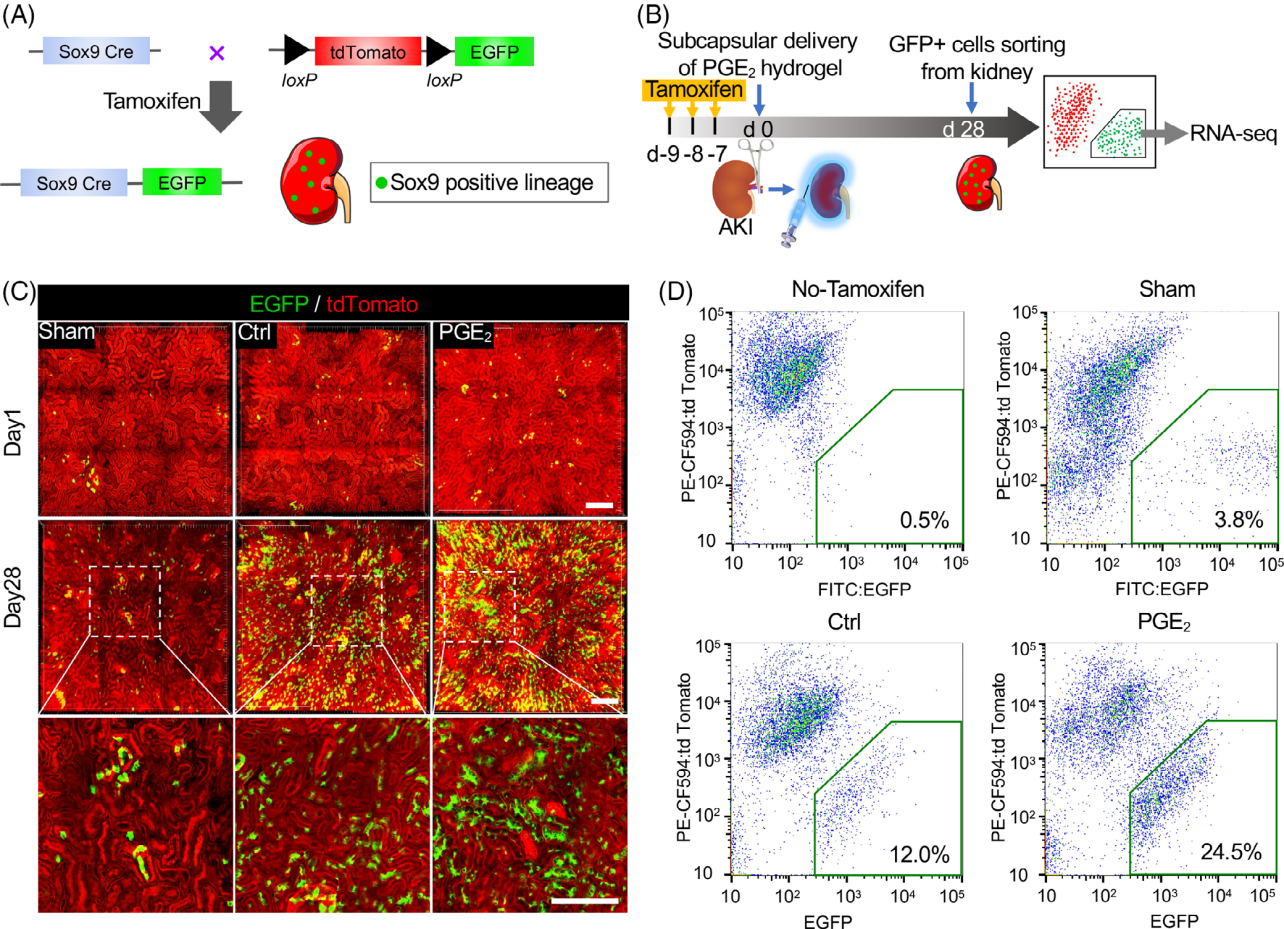
PGE_2_ activates the expansion of Sox9^+^ cells after AKI. (A) Scheme of lineage tracing of endogenous renal Sox9^+^ stem cells. (B) Diagram denoting the workflow for scRNA‐seq. (C) Representative images of intravital microscopy revealed that Sox9^+^ cell‐derived cells (green) abundantly expanded and formed renal tubule‐like structures after PGE_2_ treatment. Collagen was used as a control. Treatment with PGE_2_ promoted the proliferation of Sox9‐positive progeny cells. Scale bar, 200 μm. (D) FACS of EGFP^+^ cells in Sox9‐Cre^ERT2^; Rosa26^mTmG^ mice kidney with non‐tamoxifen, sham, collagen (control) and PGE_2_ administration at Day 28. Renal EGFP^+^ cells treated with PGE_2_ and collagen (control) were sorted with flow cytometry for scRNA‐seq. Each of the groups comprised three Sox9‐Cre^ERT2^; Rosa2^6mTmG^ transgenic mice. FACS, fluorescence‐activated cell sorting.

To decipher the transcriptional regulatory network and the cell fate decisions of Sox9^+^ cells during kidney repair, Sox9‐Cre^ERT2^; Rosa26^mTmG^ reporter mice were used (Figure 1A), and the progeny of Sox9^+^ cells (EGFP‐labelled cells) under PGE_2_ treatment were sorted for scRNA‐seq (Figure 1B). Synthesis of the PGE_2_ release matrix was performed as previously reported.^12^ Kim‐1 staining analysis revealed that PGE_2_ reduced tubular damage during early treatment of kidney injury (Figure S1A). Analysis of Sox9^+^ cell populations at various stages post‐AKI in wild‐type mice showed that Sox9^+^ cells increase rapidly in post‐AKI repair with PGE_2_ treatment (Figure S1B).

Our results revealed that EGFP^+^ cells can be tracked with high‐resolution two‐photon microscopy (TPM), which can form renal tubule‐like structures, and this can be increased by PGE_2_ (Figures 1C and S1C). However, EGFP^+^ cells were rare and most renal cells were positive for tdTomatoe in the normal adult kidney. Fluorescence‐activated cell sorting (FACS) analysis revealed that approximately 24.5% of cells were EGFP+ in kidneys treated with PGE_2_ (Figure 1D). The progeny of Sox9^+^ cells, EGFP^+^ cells, was sorted for further scRNA‐seq analysis.

### 3.2 Transcriptomic profile of the descendants of Sox9^+^ cells with PGE_2_ treatment

**FIGURE 2:**
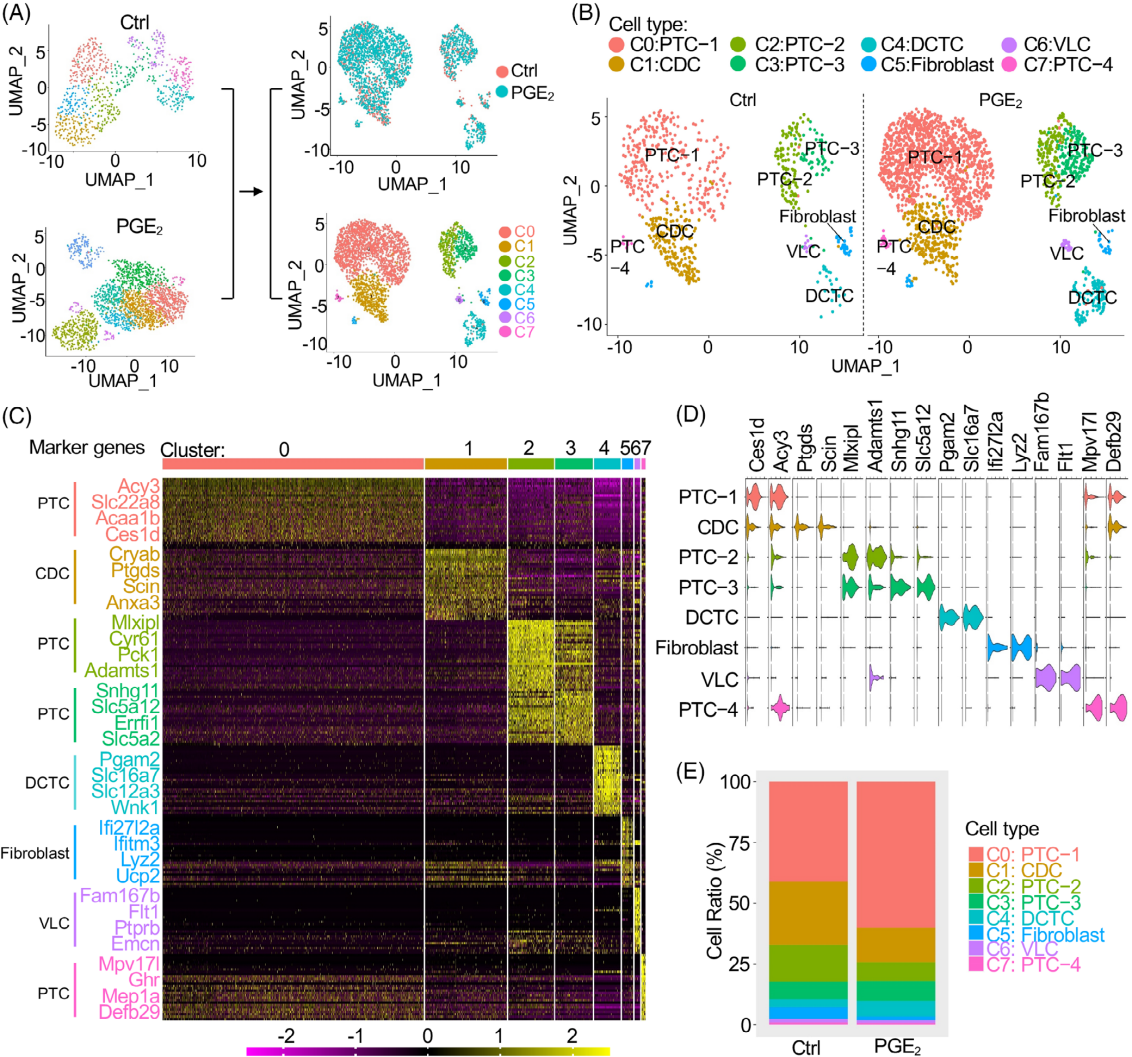
Single‐cell transcriptomic profile of the progeny of Sox9^+^ with PGE_2_ treatment. (A) Separated (left) and combined (right) UMAP plots of the progeny of Sox9^+^ stem cells (EGFP^+^ cells) on Day 28. We defined eight main cell types (C0–C7). (B) The UMAP plots reveal eight cell clusters, including proximal tubule cells (PTC‐1, PTC‐2, PTC‐3 and PTC‐4), distal convoluted tubule cells (DCTCs) and collecting duct cells (CDCs), fibroblasts and vascular endothelial‐like cells (VLCs). (C) Expression of cluster‐specific marker genes. The heatmap shows the differentially expressed genes (DEGs) in each cell cluster. Complete lists of differential genes for each cell type can be found in Table S1. (D) Violin plot of the selected gene expression in each cluster. The violin plots are coloured by cell type, and the width represents the percentage of cells expressing the marker at a given expression level in log_2_FC from cluster 0 to cluster 7. (E) Percentage distribution of each cell type. PTC, proximal tubule cell; UMAP, Uniform Manifold Approximation and Projection.

We obtained a total of 5394 cells (1363 for the control group and 4031 for the PGE_2_‐treated group), which were used to construct the sequencing library. The scRNA‐seq data showed high read depth and were mapped to approximately 1400 median genes per cell (Figure S2A). A total of eight individual clusters (C0–C7) were defined in the Seurat package^15^ to perform principal component analysis (PCA) and Uniform Manifold Approximation and Projection (UMAP) analysis from the combined data sets of PGE_2_ and control groups (Figure 2A). The eight‐cell clusters were annotated into proximal tubule cells (PTC‐1, PTC‐2, PTC‐3 and PTC‐4), distal convoluted tubule cells (DCTCs), collecting duct cells (CDCs), fibroblasts and vascular endothelial‐like cells (VLCs) (Figure 2B), which were identified by expression of cluster‐specific marker genes (Figure 2C,D **)**. Furthermore, the violin plots also revealed the expression patterns of each cluster according to cluster‐specific marker genes (Figure S2B,C). The proportion of each cell cluster revealed that PTCs were significantly increased, especially PTC‐1, whereas fibroblasts were reduced in the progeny Sox9^+^ cells treated with PGE_2_ (Figure 2E).

### 3.3 Temporal dynamics and multilineage differentiation of the Sox9 lineage

**FIGURE 3:**
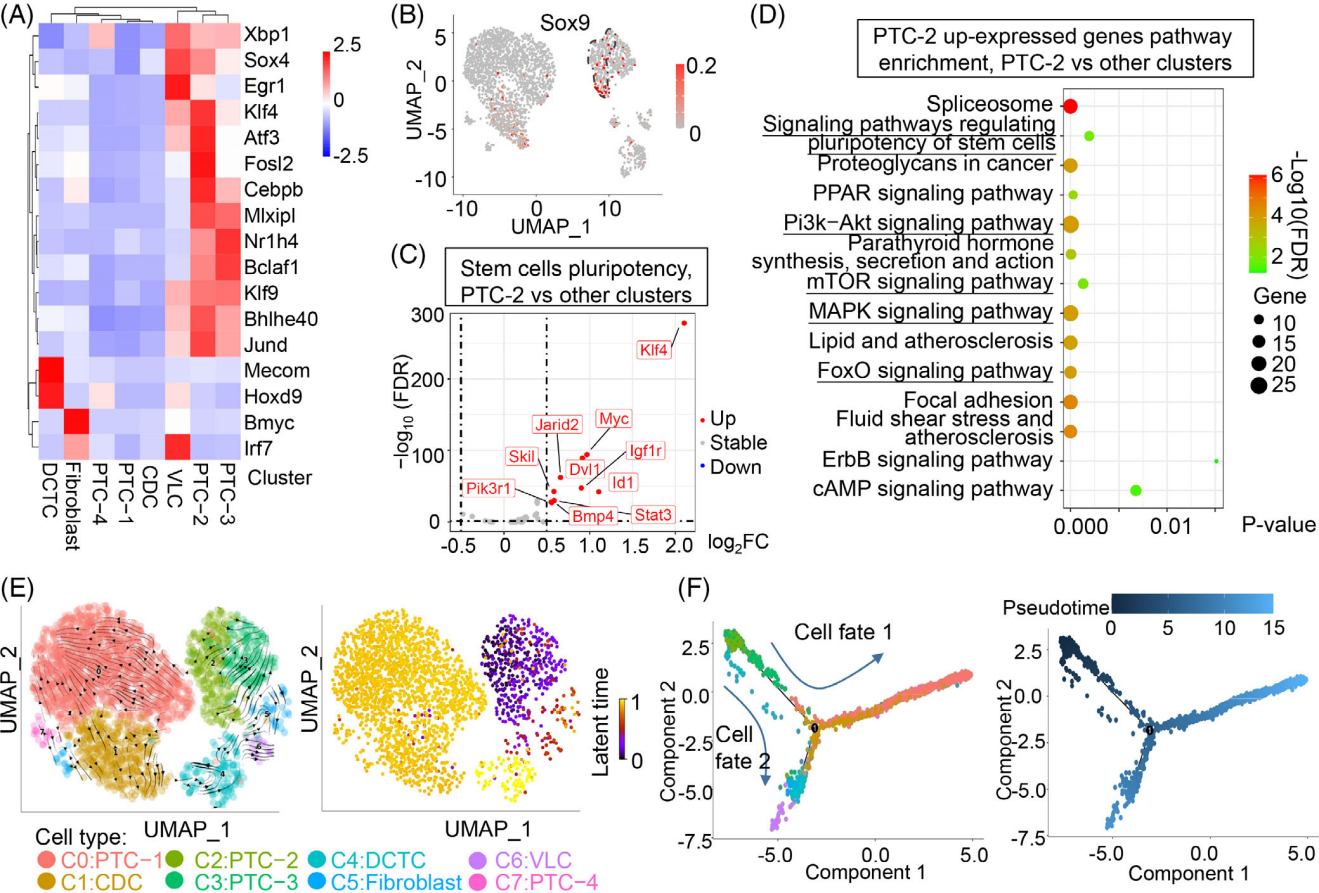
Identification of Sox9^+^ cells as endogenous renal stem cells. (A) SCENIC regulon activity heatmap depicting enriched regulons in each cell cluster of the combined data sets of the control and PGE_2_ treatment groups. Klf4, Atf3, Cebpb, Klf9 and Sox4 are related to the self‐renewal and pluripotency characteristics of stem cells, and Mlxipl, Jund, Bclaf1 and Bhlhe40 are related to cell proliferation and were enriched mainly in PTC‐2. (B) Feature plots showing the average expression (colour‐scaled) of Sox9 in each cell cluster, and PTC‐2 manifested a major enrichment of Sox9. The area enclosed by the black dotted lines represents the PTC‐2 cell cluster. (C) Volcano plot of cells in PTC‐2 versus the other clusters. Dashed lines represent the threshold used for the fold change and FDR (|log_2_FC| > 0.5, FDR < 0.05). FDR, false discovery rate. The complete lists of DEGs between PTC‐2 and other clusters in the combined data sets are shown in Table S2. (D) Significantly enriched KEGG terms based on upregulated DEGs in PTC‐2 compared to all other clusters. log_2_FC >0.5, *p* value <0.05. Those marked in red were related to the function and characteristics of stem cell‐related signalling pathways. The complete lists of upregulated gene‐related pathways between PTC‐2 and other clusters in the combined data sets are listed in Table S3. (E) RNA velocity analysis in the combined data sets of the control and PGE_2_ treatment groups. Latent time represents the pseudo‐time of the RNA velocity. (F) Pseudo‐time trajectory analysis of each cell cluster in combined data sets using Monocle 2. Cells are colour‐coded by cell clusters (left) or pseudo‐time (right). Pseudo‐time is a simulated time variable that indicates the relative position that a cell takes in a lineage. DEG, differentially expressed gene; PTC, proximal tubule cell.

To explore the mechanism of kidney repair, we sought to identify transcription factors related to kidney regeneration with single‐cell regulatory network inference and clustering (SCENIC),^16^ and 17 transcription factor regulons were enriched in the combined data sets (Figure 3A). The PTC‐2 cluster manifested an enrichment of Klf4, Atf3, Cebpb, Klf9 and Sox4, which were related to the characteristics of self‐renewal and pluripotency of stem cells. Furthermore, transcription factors known to be involved in cell proliferation, including Mlxipl, Jund, Bclaf1 and Bhlhe40, were detected in the PTC‐2 cluster (Figures 3A and S3A). The feature plots of the progeny of Sox9^+^ cells revealed that most descendants of EGFP^+^ cells no longer expressed Sox9. However, cells from the PTC‐2 cluster showed significant enrichment of Sox9 expression (Figure 3B). To explore the functional status of Sox9^+^ cells in the PTC‐2 cluster, we performed DEG analysis between PTC‐2 and all other cells in the combined subset. As expected, there was a significantly increased expression of genes related to pluripotency of stem cells, such as Myc, Klf4, Dvl1, Jarid2, lgf1r, Acox1, Id1, Stat3, Skil and Bmp4, in the PTC‐2 cell cluster (Figure 3C). Further analysis with the Path View package as reported before^17^ and Kyoto Encyclopedia of Genes and Genomes (KEGG) enrichment revealed that the function and characteristics of stem cell pluripotency signalling pathways, Pi3k‐Akt,^18^ mTOR,^19^ FoxO^20^ and Mapk signalling pathway^21^ were enriched in PTC‐2 cells (Figures 3D and S3B–F). Furthermore, based on the Physical Core database, a protein–protein interaction network of candidate marker genes in PTC‐2 was constructed by Metascape software, which revealed that these pathway proteins exhibited high interactions (Figure S4A). Gene ontology (GO) analysis revealed an increase in tissue morphogenesis, epithelial cell differentiation, developmental growth and regulation of growth biological processes in PTC‐2, which are needed for tissue repair and regeneration (Figure S4B,C). These results suggested that Sox9^+^ cells in PTC‐2 were the cluster that retained stem cell properties, which might play an important role in kidney regeneration.

To explore the directionality of the pseudo‐time trajectory, we performed RNA velocity analysis to estimate the expression of spliced and unspliced mRNAs in the trajectory.^22^ Potential directionality of cell state transitions was observed between PTC‐2 and intra‐subpopulations, which indicated that these subpopulations recapitulated the early to late transition with latent time and showed positive velocity from PTC‐2 to PTC‐1, PTC‐3, CDC and fibroblasts (Figures 3E and S4D). Furthermore, cluster‐specific differential velocity analysis of combined data sets revealed that kidney development, tube morphogenesis and regulation of epithelial cell proliferation‐related genes were predicted to be dynamically upregulated in PTC‐2 (Figure S4E). To further estimate the lineage relationships among renal cells, we performed a Monocle analysis^23^ in the combined data sets of PGE_2_ and control groups. The results suggested that PTC‐2 can differentiate into seven other types of renal cells along the two directions of cell fate of the pseudo‐time line (Figure 3F). For Sox9^+^ cell fated branch 1 (cell fate 1 in Figure 3F, gene set 1 in Figure S4F), functional genes involved in the catabolic process of small molecules and anion transmembrane transport were provoked in PTC‐1, CDC and PTC‐4 late along the pseudo‐time, reflecting the differentiation tendency of proximal epithelial cells and collecting duct cells. Cells from Sox9^+^ cell fated branch 2 (cell fate 2 in Figure 3F, gene sets 2 and 3 in Figure S4F) enriched GO terms of ‘sodium ion homeostasis, chloride ion homeostasis, phosphagen metabolic process, fibroblast proliferation’, all of which were identified as markers of DCTC, fibroblast and VLC, thus deciphering cell fate decisions during tubule cell differentiation of the Sox9 lineage in kidney regeneration.

### 3.4 The dynamics of the Sox9 lineage in AKI with PGE_2_ treatment

**FIGURE 4:**
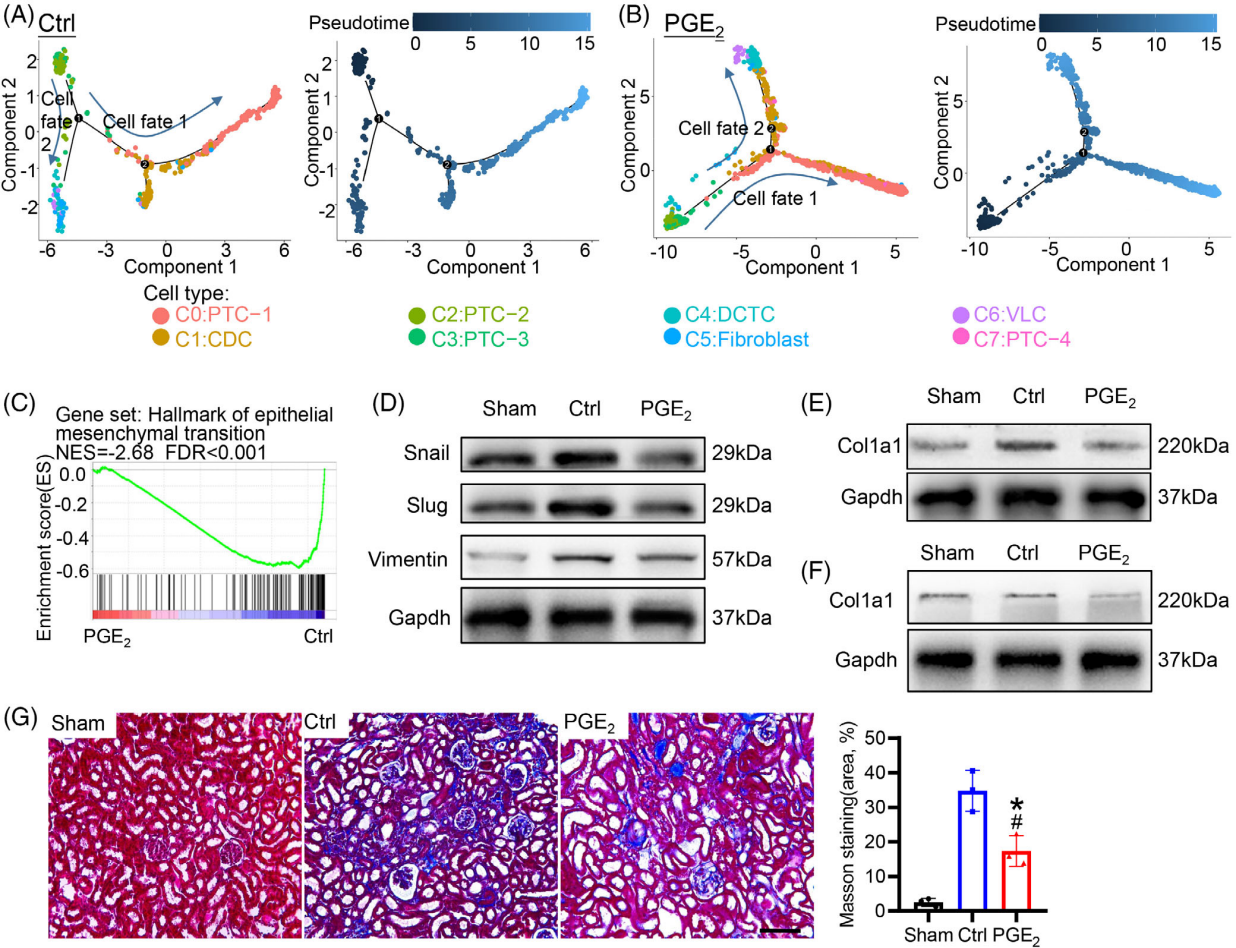
PGE_2_ promotes renal proximal tubular epithelial cell differentiation of Sox9^+^ cells and inhibits the development of kidney fibrosis. (A, B) Differentiation pseudo‐time of all lineage cells in the control group and the PGE_2_ treatment group. Treatment with PGE_2_ delayed the differentiation of PTC‐2 cells into fibroblasts. (C) GSEA enrichment plots from the hallmark gene data set associated with epithelial–mesenchymal transition in the PGE_2_‐treated group compared to the control, suggesting that PGE_2_ treatment reduced the progression of epithelial–mesenchymal transition. (D) Immunoblotting analysis of epithelial–mesenchymal transition markers (Snail, Slug and Vimentin) in the kidney on Day 28 after AKI. (E) Immunoblotting analysis of Col1a1 in the kidney on Day 28 after AKI. (F) Western blotting analysis of Col1a1 expression in Sox9^+^ cells cultured on non‐coated, COL or PGE_2_ matrix at 48 h. Sox9^+^ cells were isolated from Sox9‐Cre^ERT2^; Rosa26^mTmG^ mice treated with tamoxifen for 3 days. (G) Representative images of Masson trichrome staining revealed that PGE_2_ treatment reduced kidney fibrosis. AKI, acute kidney injury; PTC, proximal tubule cell. Scale bars, 100 μm. *n* = 3 mice for each group, *p value <0.05 versus sham; #*p* value <0.05 versus control.

To further evaluate the differentiation of the Sox9 lineage in the process of kidney regeneration after AKI, a Monocle trajectory was applied to infer the developmental timeline. Trajectory analysis revealed a gradual transition from PTC‐2 to PTC, CDC, DCTC or fibroblast in the cell fate 1 and fate 2 pseudo‐time line, but PGE_2_ treatment exhibited different kinetics of Sox9 lineage differentiation (Figure 4A,B). By activation of PGE_2_, most cells from the PTC‐2 cluster undergoing differentiation followed by cell fate 1 and cell fate 2 were identified as PTC, DCTC and CDC, whereas fibroblast differentiation was detected in the control group. Additionally, the associated genes in anion transport, chloride ion homeostasis, small‐molecule biosynthetic process, and catabolic fatty acid process were enriched during the differentiation of PTC‐2. Notably, genes associated with positive regulation of fibroblast growth factor production (Aif1), fibroblast markers (such as Ucp2, Ms4a6c and Lyz2), and regulation of lymphocyte proliferation were progressively upregulated in cell fate 2 of the control group, whereas they were absent in the PGE_2_‐treated group (Figure S5A,B). These results suggested that PTC‐1 starts from the PTC‐2 cell population, which is promoted by PGE_2_, but the fibroblast differentiation of PTC‐2 was inhibited with PGE_2_ treatment.

The epithelial–mesenchymal transition (EMT) has been described as a mechanism by which injured renal tubular cells undergo mesenchymal cell phenotype changes that contribute to renal fibrosis.^24^ Gene set enrichment analysis (GSEA) indicated that PGE_2_ treatment effectively inhibited the progression of EMT and the development of fibrosis (Figure 4C), which was further confirmed by Western blot analysis of Snail, Slug and Vimentin (EMT markers) (Figure 4D). GSEA for each cell cluster found after PGE_2_ treatment, there was a significant inhibition of EMT in the majority of cells, including PTC, CDC, and a small number of fibroblasts (Figure S5C). Additionally, pathological collagen deposition is a hallmark of renal fibrosis, and we found that PGE_2_ treatment inhibited the expression of Col1a1 in Sox9^+^ cells and kidneys after AKI (Figure 4E,F), which was further confirmed by Masson trichrome staining (Figure 4G). Overall, these results suggested that PGE_2_ could promote the differentiation of Sox9^+^ cells into renal proximal tubular epithelial cells and inhibit the development of fibrosis.

### 3.5 PGE_2_ promotes proximal tubule repair by activating Sox9^+^ cells

**FIGURE 5:**
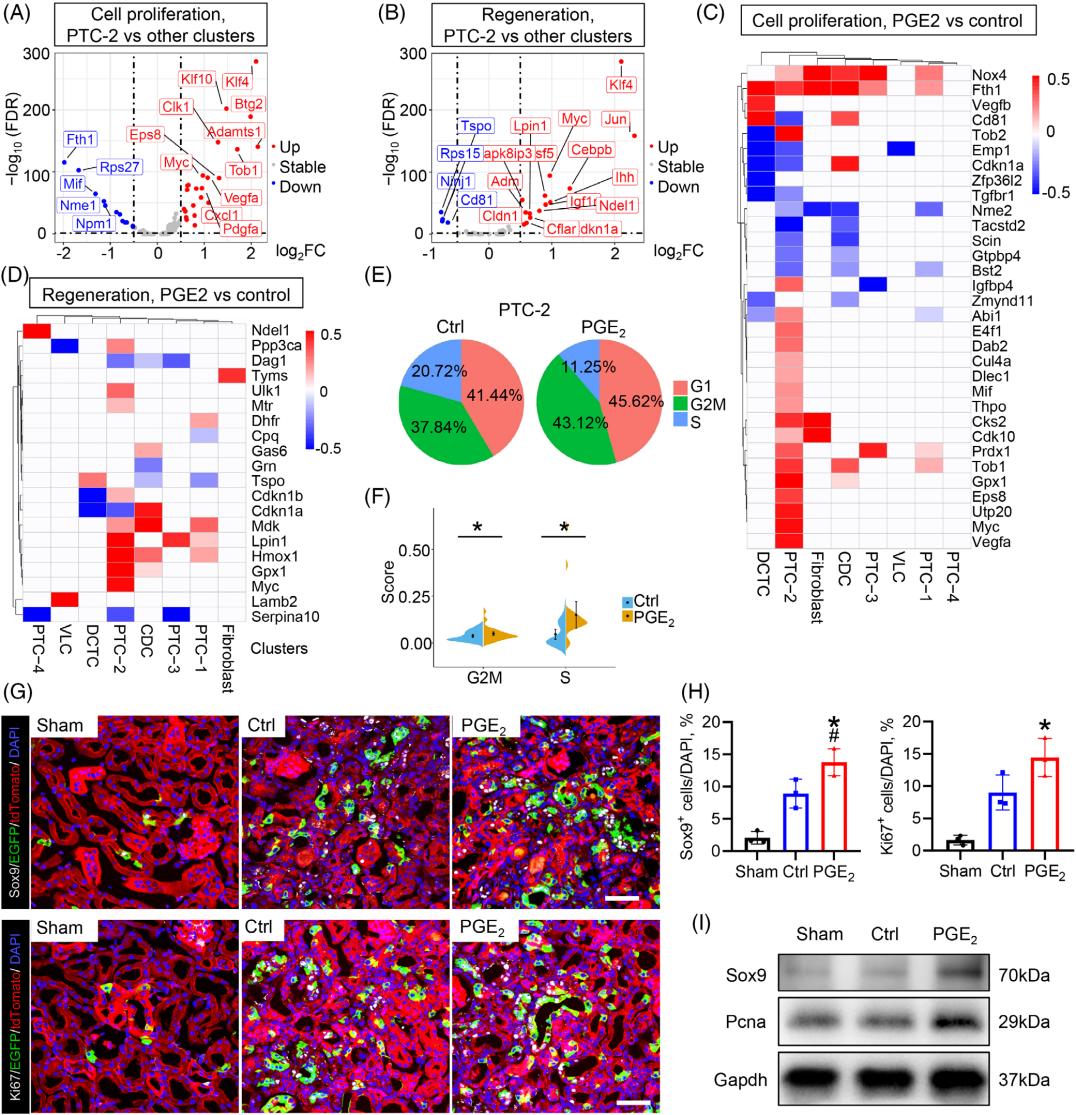
PGE_2_ promoted Sox9^+^ cell proliferation and maintained stem cell pluripotency to orchestrate proximal tubule repair. (A, B) Volcano plot of cells in PTC‐2 versus the other clusters. The listed differentially expressed genes belong to the ‘Cell proliferation’ and ‘Regeneration’ gene sets, respectively. Dashed lines represent the threshold used for the fold change and FDR (|log_2_FC| > 0.5, FDR < 0.05). FDR, false discovery rate. The complete list of DEGs between PTC‐2 and other clusters in the combined data sets is shown in Table S2. (C) The heatmap shows the relative gene expression of DEGs enriched in functional blocks related to cell proliferation in each type comparing the PGE_2_ and control groups. The complete lists of DEGs between PGE_2_ and Ctrl in all cell clusters are listed in Table S5. *p* Value <0.05. (D) The heatmap shows the relative gene expression of DEGs enriched in functional blocks related to cell regeneration in each type. *p* value <0.05. (E) The percentage of cells that were present in the cell cycle analysis revealed that PGE_2_ could activate the proliferation of PTC‐2 cells. (F) Violin plots show the G2/M and S‐score of cycling basal cells and cycling intermediate cells in PTC‐2. *p value <0.05. (G) Representative images for colocalization analysis of Sox9 (grey), Ki67 (grey) and Sox9‐Cre^ERT2^‐activated EGFP fluorescence in kidneys. Scale bar, 50 μm. (H) Quantification analysis revealed that PGE_2_ could promote the expression of Vegfa and Ki67 in the progeny of Sox9^+^ cells. Data are expressed as mean ± SD; *n* = 3 mice for each group, *p value <0.05 versus sham, #*p* value <0.05 versus control. (I) Western blot analysis of Sox9 and Pcna in the kidney on Day 28 after AKI. AKI, acute kidney injury; DEG, differentially expressed gene.

Next, we investigate the expression levels of genes related to cell proliferation and regeneration among the different cell types. Upregulation of genes related to cell proliferation and regeneration was expressed mainly in PTC‐2, followed by PTC‐3 (Figures 5A,B and S6A,B, Table S4), suggesting that PTC‐2 represented the main population of proliferation and regeneration. Notably, the expression of proliferation and regeneration‐related genes, mainly in PTC‐2 cells, increased with PGE_2_ treatment (Figure 5C,D). In addition, we evaluated the signatures of different cycling phases in PTC‐2 and observed that the G2/M score of PTC‐2 was significantly higher in the PGE_2_‐treated group, and the percentage of cells in the proliferative state (G2/M phase) in PTC‐2 was significantly higher than that in the control group (Figure 5E,F). These results suggested that PGE_2_ activated the proliferation and regeneration of Sox9^+^ cells, thereby reconstructing renal structures in kidney repair. Interestingly, DEG analysis revealed that PGE_2_ treatment increased the expression of gene sets related to stem cell characteristics, especially Myc expression, in PTC‐2 cells compared to control cells (Figure S6C, Table S4).

To confirm the activating effect of PGE_2_ on the proliferation and stem cell pluripotency of endogenous stem cells, further immunofluorescence analysis confirmed that increased expression of Sox9 and Ki‐67 could be detected in EGFP^+^ cells treated with PGE_2_ (Figure 5G,H). In addition, the histological results were further confirmed by Western blot analysis of Sox9 and Pcna (Figure 5I). Immunostaining results revealed that the double‐positive Sox9 and Ki‐67 cells increased with PGE_2_ treatment (Figure S6D). Taken together, these findings suggested that activated Sox9^+^ cells can boost the differentiation of proximal tubule cells by increasing Vegfa secretion, which can be promoted by PGE_2_.

### 3.6 PGE_2_ promotes Sox9^+^ cell homeostasis through Pi3k‐Akt signalling pathway

**FIGURE 6:**
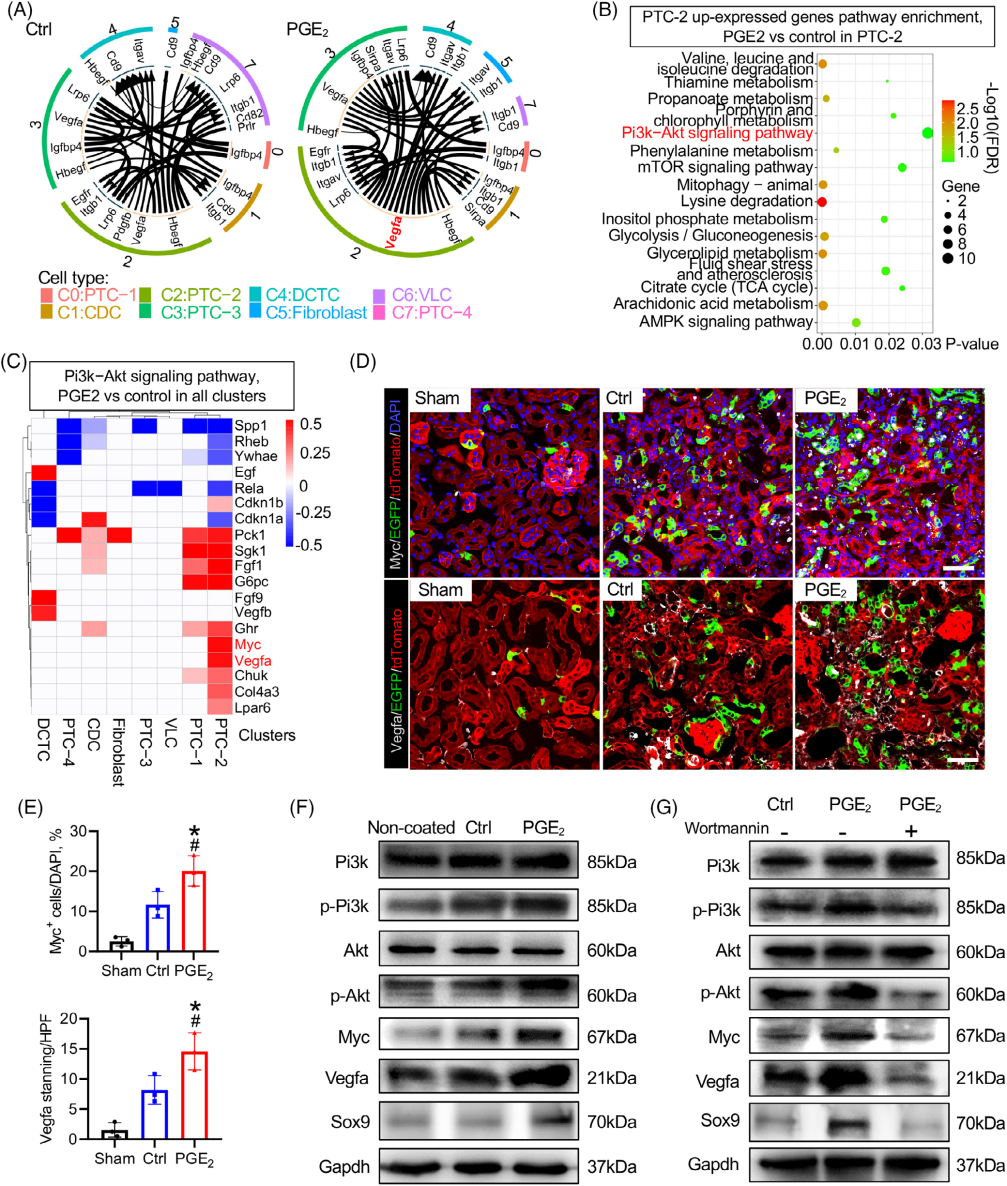
PGE_2_ maintains the pluripotency of Sox9^+^ cells and induces Vegfa production by activating the Pi3k‐Akt signalling pathway. (A) Lines represent growth factor‐mediated interactions between cell types in the kidney, showing the top 30 upregulated ligand–receptor interactions. The ligands are labelled pink, and the receptors are labelled dark blue. The width of the arrow represents the expression level (log_2_FC) of the ligand, while the width of the arrowhead represents the expression level (log_2_FC) of the receptor. The crosstalk of growth factor‐mediated intercellular communication among cell types in the control and PGE_2_ treatment groups is listed in Table S6. (B) KEGG pathway enrichment analysis of upregulated DEGs in PTC‐2. The number of genes upregulated related to the Pi3k‐Akt signalling pathway was the highest among the upregulated genes. Complete lists of upregulated genes upon PGE_2_ treatment for the cell clusters of PTC‐2 are listed in Table S7. (C) The heatmap of DEGs revealed the upregulation of Pi3k‐Akt signalling pathway activation‐related genes in the PTC‐2 cluster with PGE_2_ treatment. (D, E) Representative images and quantification for colocalization analysis of anti‐Myc (grey) and anti‐Vegfa (grey) immunostaining and Sox9‐Cre^ERT2^‐activated EGFP fluorescence in kidneys. Scale bar, 50 μm. *n* = 3 mice for each group, *p value <0.05 versus sham; #*p* value <0.05 versus control. (F, G) Western blotting analysis of Pi3k, p‐Pi3k, Akt, p‐Akt, Myc and Vegfa expression. Sox9^+^ cells were isolated from Sox9‐Cre^ERT2^; Rosa26^mTmG^ mice treated with tamoxifen for 3 days and cultured in non‐coated collagen and PGE_2_ matrix with/without the Pi3k‐Akt inhibitor (wortmannin, 1 μM) for 48 h. Gapdh was used as a loading control. Quantification of Western blotting analysis can be found in Figure S8A,B. DEG, differentially expressed gene; KEGG, Kyoto Encyclopedia of Genes and Genomes; PTC, proximal tubule cell.

Tissue repair and regeneration rely on intricate and dynamic interactions between multiple types of functional cells. To examine whether PGE_2_ regulates cell proliferation or stem cell maintenance through intercellular communication, we analysed differences in growth‐related ligand receptors in kidneys treated with PGE_2_. Notably, data of the ligand–receptor pair indicated that growth‐related communication, such as Vegfa, increased significantly with PGE_2_ treatment (Figures 6A and S7A,B). Vegfa activation can protect cells from hypoxia, a mechanism that in PTCs has been proposed to be nephroprotective.^25^ Additionally, the DEG analysis revealed that a high expression of Vegfa receptor‐related genes was observed in PTC‐2 (Figure S7C).

To explore the regulatory effect of PGE_2_ on cells of the PTC‐2 cluster, we performed KEGG and DEG analysis, and the results indicated that the phosphoinositide 3‐kinase (Pi3k)‐Akt signalling pathway was enriched in PTC‐2 cells treated with PGE_2_ **(**Figure 6B). Several studies have shown that Myc regulates the cell cycle, DNA replication, ribosome biogenesis, and metabolism for stem cell proliferation and stem cell self‐renewal.^26^, ^27^ The Pi3k‐Akt signalling pathway, including Myc and Vegfa, was significantly upregulated in PTC‐2 cells (Figure 6C). These results suggested that PGE_2_ promotes Myc expression in Sox9^+^ cells by activating the Pi3k‐Akt pathway, which in turn increases Vegfa secretion.

Further immunohistology results confirmed this observation, and data revealed that upregulated Myc and Vegfa could be detected in EGFP^+^ cells by PGE_2_ treatment (Figure 6D,E). Western blot analysis revealed that PGE_2_ stimulated Myc, Vegfa, and Sox9 through the Pi3k‐Akt‐mediated pathway (Figures 6F and S8A). As expected, inhibition of Pi3k by wortmannin significantly reduced phospho‐Akt levels. Moreover, a significant downregulation of Myc, Vegfa and Sox9 protein levels in Sox9^+^ cells was accompanied by inactivation of Akt (Figures 6G and S8B).

### 3.7 PGE_2_ maintains the metabolic homeostasis of PTCs

**FIGURE 7:**
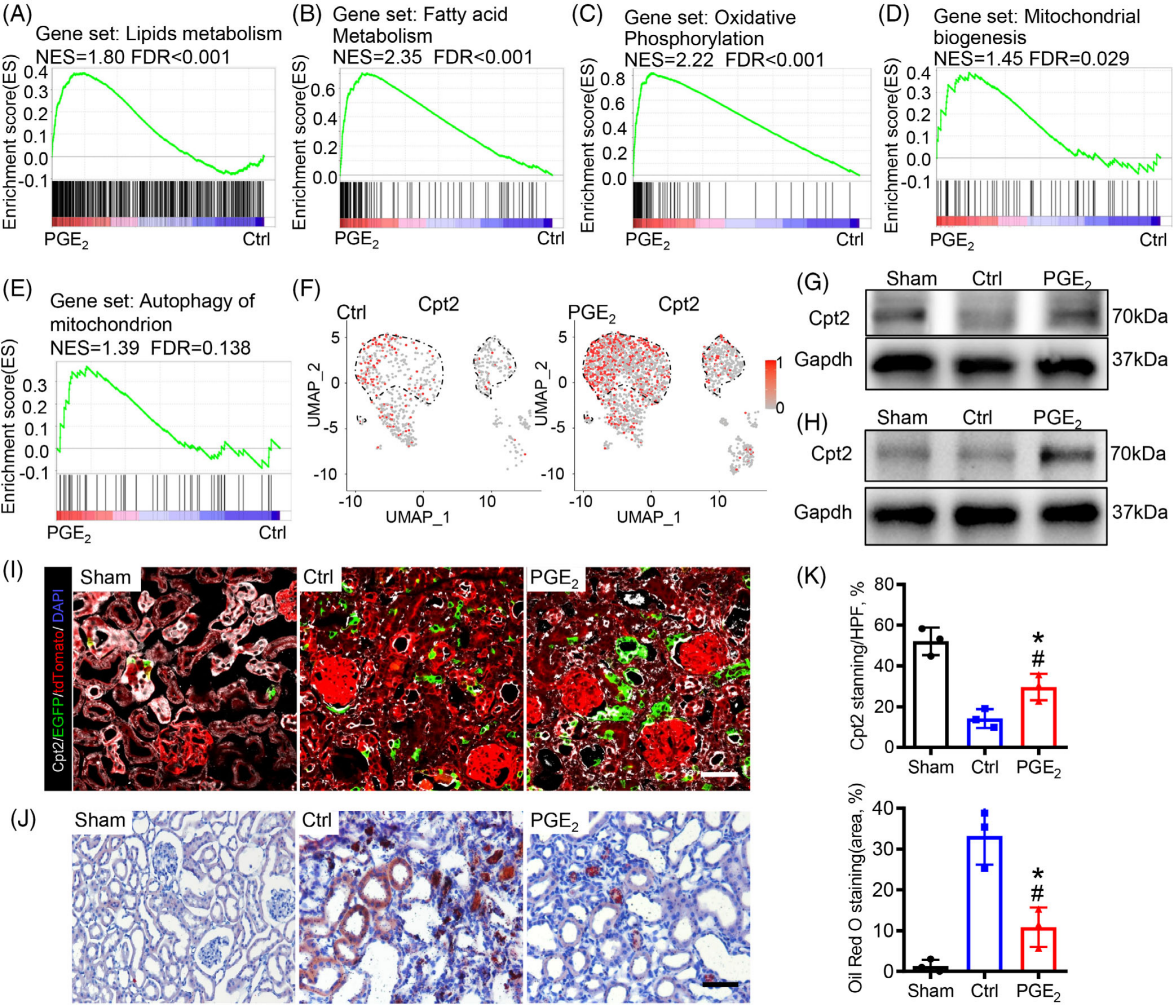
PGE_2_ treatment maintains the metabolic homeostasis of proximal tubular epithelial cells. (A–C) GSEA enrichment plots of gene data sets associated with lipid metabolism, fatty acid metabolism and oxidative phosphorylation in all PTCs of PGE_2_‐treated kidneys compared to the control. (D, E) GSEA enrichment plots of gene data sets associated with energy metabolism homeostasis. Compared to the control group, mitochondrial biogenesis and mitophagy were mainly enriched in PGE_2_‐treated renal PTCs. (F) U‐map plot, colour coded for the expression of the Cpt2 genes (grey to red), showing the increased expression of Cpt2 in PTCs after PGE_2_ treatment. The area enclosed by the black dotted lines represents all the proximal tubule cell clusters. (G) Immunoblotting analysis of Cpt2 in the kidney on Day 28 after AKI. (H) Western blotting analysis of Cpt2 expression in Sox9^+^ cells cultured on non‐coated, COL or PGE_2_ matrix at 48 h. (I) Cpt2 staining of the kidneys of Sox9‐Cre^ERT2^; Rosa26^mTmG^ mice groups with sham, control or PGE_2_ treatment on Day 28 after AKI. Scale bar, 50 μm. (J) Oil‐red O staining of the kidneys of Sox9‐Cre^ERT2^; Rosa26^mTmG^ mice groups with sham, control or PGE_2_ treatment on Day 28 after AKI. Scale bar, 50 μm. (K) Respective quantification of the Cpt2 staining area (up) and Oil Red O staining (down). One‐way ANOVA with the Tukey post hoc test was used for statistical analysis. AKI, acute kidney injury; GSEA, gene set enrichment analysis; PTC, proximal tubule cell. Data are expressed as mean ± SD; *n* = 3 mice for each group, *p value <0.05 versus sham; #*p* value <0.05 versus control.

Kidney injury primarily targets tubular epithelial cells, especially the highly metabolically active proximal tubular segment.^28^ To determine whether PGE_2_ could regulate the metabolic homeostasis of proximal tubular epithelial cells through intercellular communication, GO biological process analysis revealed that associated genes were enriched in the monocarboxylic acid metabolic process, the generation of precursor metabolites and energy and the dicarboxylic acid metabolic process in all PTCs with PGE_2_ treatment (Figure S9A). Furthermore, GSEA results highlighted a strong enrichment of genes related to lipid metabolism, fatty acid metabolism and oxidative phosphorylation in all PTCs treated with PGE_2_ (Figure 7A–C). Maintenance of energy metabolism homeostasis is achieved by coordinating mitochondrial biogenesis and mitochondrial autophagy, which promote cell survival and stress resistance.^29^ Related genes involved in mitochondrial homeostasis and autophagy were highly enriched in renal proximal tubule cells after PGE_2_ treatment (Figure 7D,E), indicating that PGE_2_ maintains mitochondrial renewal and homeostasis after kidney injury, thus maintaining homeostasis of lipid metabolism.

Carnitine palmitoyltransferase 2 (Cpt2), the rate‐limiting enzyme involved in the transport of fatty acids to mitochondria for beta‐oxidation, is widely recognized as an emerging therapeutic target.^28^ As expected, DEG analysis revealed that PGE_2_ increased the expression of Cpt2 in PTCs (Figure 7F), which was further confirmed by Western blot analysis of Cpt2 (Figure 7G). Additionally, we found that PGE_2_ treatment significantly upregulated Cpt2 expression in Sox9^+^ cells (Figure 7H). These results were validated by the increase in the expression of Cpt2 detected by immunofluorescence in PGE_2_‐treated kidneys compared to the control (Figure 7I,K). To examine lipid metabolism, we performed Oil Red O staining. We found that lipid droplets increased significantly on Day 28 after AKI, suggesting a persistent accumulation of lipids in the proximal tubule. With PGE_2_ treatment, lipid accumulation was significantly reduced (Figure 7J,K). These results suggested that activated Sox9^+^ cells exhibited superior outcomes in restoring mitochondrial β‐oxidation, promoting mitochondrial biosynthesis, and maintaining metabolic homeostasis of PTCs through intercellular communication of Vegfa.

### 3.8 PGE_2_ administration prevents the progression of AKI to CKD

**FIGURE 8:**
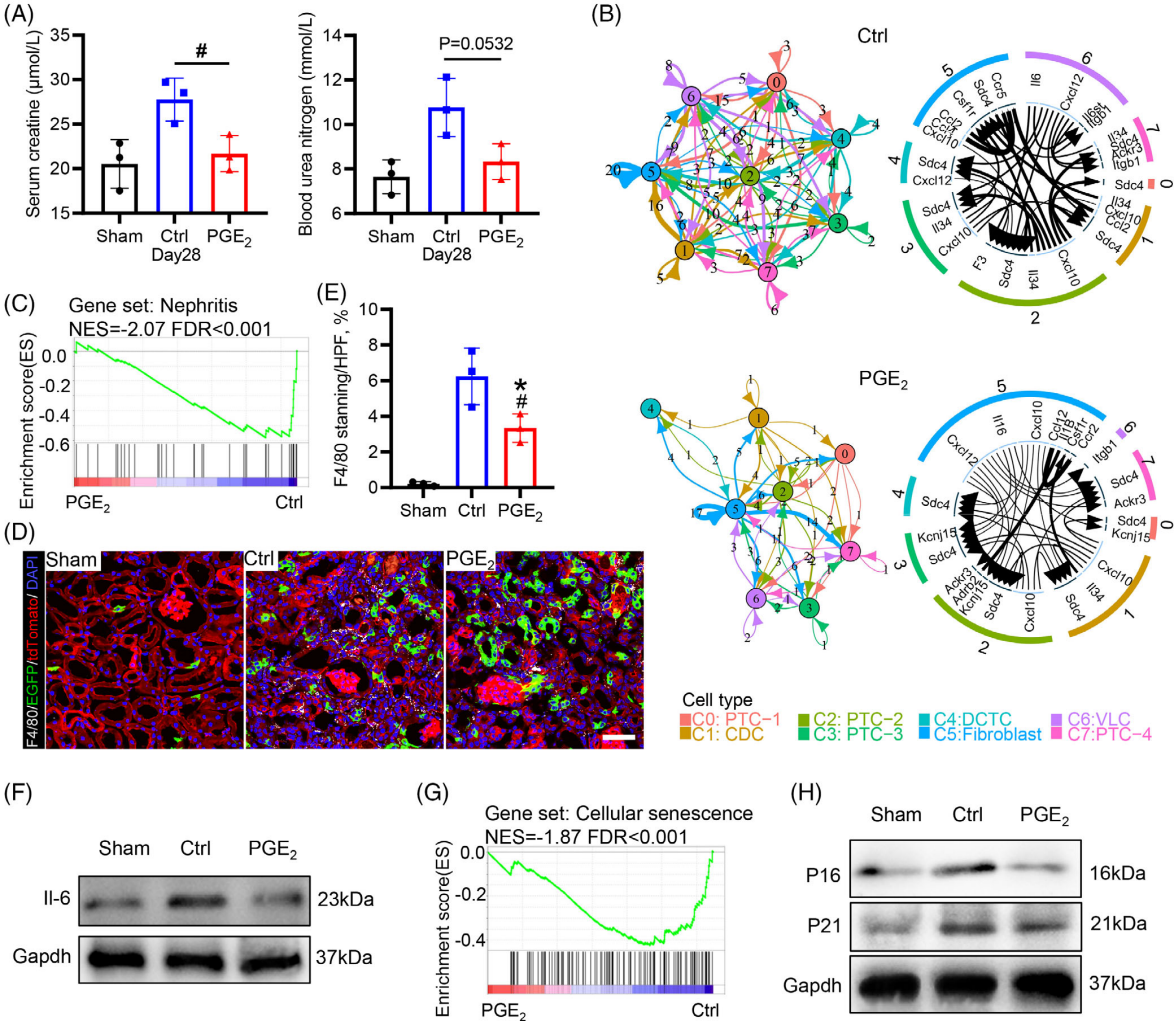
PGE_2_‐mediated activation of Sox9^+^ cells improves recovery of kidney function and inhibits transformation to chronic kidney disease. (A) Serum creatinine (top panel) and blood levels of urea nitrogen (bottom panel) were measured on Day 28 after AKI. (B) Network plot showing the number of cytokine ligand–receptor interactions detected between each of the two different cell types and/or within the same cell type. The crosstalk of cytokine‐mediated intercellular communication among cell types in control and PGE_2_‐treated kidneys is listed in Table S8. (C) GSEA enrichment plot of the gene data set associated with nephritis in PGE_2_‐treated kidneys compared to control kidneys. (D, E) Representative images and quantification of the colocalization analysis of anti‐F4/80 immunostaining (grey) and Sox9‐Cre^ERT2^‐activated EGFP fluorescence in kidneys, which indicates that PGE_2_ treatment reduced the long‐term infiltration of inflammatory cells after AKI. Scale bar, 50 μm. *n* = 3 mice for each group, *p value <0.05 versus sham; #*p* value <0.05 versus control. (F) Immunoblotting analysis of pro‐inflammatory factor (Il‐6) in the kidney on Day 28 after AKI. (G) GSEA enrichment plot of the gene data set associated with cellular senescence in PGE_2_‐treated kidneys compared to control kidneys. (H) Immunoblot analysis of cellular senescence markers (P16 and P21) in the kidney on Day 28 after AKI. AKI, acute kidney injury; GSEA, gene set enrichment analysis.

To assess the therapeutic effects of PGE_2_ on renal functional recovery after AKI, we tested BUN and sCr on Day 28, and the results revealed that PGE_2_ accelerated kidney functional recovery, as shown by the lower values of BUN and sCr (Figure 8A). GO analysis of the signatures of each group demonstrated that PGE_2_‐mediated activation of Sox9^+^ cells could play a role in improving renal function after injury, as evidenced by significantly enriched biological process terms associated with renal physiological function, such as sodium ion transport, anion homeostasis, inorganic ion homeostasis, anion transmembrane transport renal sodium ion transport and regulation of body fluid level, which were absent in the control (Figure S9B). Furthermore, the control group was significantly enriched for signatures of nephrotic syndrome in the kidney after acute injury (Figure S9C).

Next, we also analysed the differences in the cytokine ligand receptors, and we observed a significant decrease in intercellular communication of proinflammatory factors for each cell cluster under PGE_2_ treatment (Figure 8B). For example, the number of proinflammatory chemokine ligand/receptor pairs in all cell types decreased from 172 to 87. Furthermore, the control group was significantly enriched for signatures of nephritis **(**Figure 8C). F4/80 has been used widely as a marker for macrophages, anti‐F4/80 immunostaining to assess renal inflammation showed sparse macrophage infiltration within the renal parenchyma after AKI, which was reduced with PGE_2_ treatment (Figure 8D,E). Further western blot analysis of inflammatory factors showed that the expression of Il‐6 decreased significantly with PGE_2_ treatment after AKI (Figure 8F). Additionally, recent studies demonstrated that pro‐inflammatory cytokines could induce cellular senescence,^30^ we analysed the differences in the enrichment of cellular senescence, and the PGE_2_ treatment group was significantly decreased for signatures of cellular senescence in the kidney after AKI (Figure 8G), which was further confirmed by Western blot assay of cellular senescence markers (P16 and P21) (Figure 8H). Collectively, PGE_2_‐mediated activation of Sox9^+^ cells may play an important role in promoting functional recovery from kidney injury and inhibiting the transformation into chronic kidney disease by promoting the restoration of the structure and metabolism of the proximal tubules.

## 4 DISCUSSION

Here, we combined single‐cell transcriptomics and lineage tracing to build a comprehensive and unbiased Sox9^+^ cell differentiation atlas during the post‐repair phase of AKI. Cell composition, cellular interactions, developmental trajectory and functional states of Sox9^+^ cell progeny with PGE_2_ activation were explored and systematically compared. Sox9 cell lineage tracing with the scRNA‐Seq approach could well link the state of Sox9^+^ cells to the end fate and further establish sister cell relationships. These findings will help us to understand the regulatory mechanisms of endogenous renal stem cell stimulation in‐depth and provide potential targets for clinical therapies for AKI.

In the present study, regenerative dynamics analysis indicated more genes that promote the epithelial tube, epithelium morphogenesis, and proximal tubule function by activating Sox9^+^ cells. Vegfa has been reported to play an important role in the proliferation of PTCs, the maintenance of tubulointerstitial integrity, and the response to AKI.^31^, ^32^ Activation of Vegfa may protect cells from hypoxia, a mechanism that in proximal tubular epithelial cells has been proposed to be nephroprotective.^25^ Furthermore, the interaction between impaired Vegf production caused by damaged proximal tubules and peritubular rarefaction may contribute to the transition from AKI to CKD.^33^ Our findings indicated that PGE_2_ promoted the expansion of Sox9^+^ cells and stimulated Vegfa expression in Sox9^+^ cells to enhance their proliferation and regeneration capacity. Boosted the formation of proximal tubule cells by increasing the secretion of Vegfa from activated Sox9^+^ cells to participate in intercellular communication. Furthermore, we also found that PGE_2_ promoted Myc expression by activating the Pi3k‐AKt signalling pathway, and previous studies have shown that Myc is a downstream transcription factor produced by activating the Pi3k‐Akt pathway,^34^ which can promote the expression and release of Vegfa.^35^

In addition, resident endogenous stem cells produce daughter cells in the progress of endogenous tissue regeneration and maintain stemness to renew themselves, while the other cells differentiate into a specific cell to conduct endogenous repair.^36^ Therefore, maintaining stem cell characteristics is important to keep resident endogenous stem cells in an undifferentiated state for tissue homeostasis, repair, and regeneration. In early embryogenesis, Myc genes are essential for murine embryonic stem cell pluripotency and self‐renewal.^37^, ^38^ Our findings indicated that PGE_2_ was conducive to maintaining stem cell characteristics by promoting Sox9 expression through the Pi3k‐Akt‐Myc signalling pathway in the injured kidney. Exogenous PGE_2_ appeared to be an especially robust enhancer of Sox9^+^ cell proliferation, while administration of PGE_2_ led to a subtle but significant increase in regeneration in the AKI model. Additionally, our results revealed that not all renal tubular cells are labelled by EGFP, indicating that newly generated Sox9^+^ cells may play similar regenerative properties. Previous studies have shown that some renal tubular cells could dedifferentiation with the characteristics of endogenous progenitor cells, and participate in the repair of the injured renal tubular cells.^39^ Therefore, PGE_2_ may also be involved in the treatment of kidney injury by regulating the proliferation and differentiation of these newly generated Sox9^+^ stem cells.

Proximal tubule cells are one of the most energy‐demanding cells and efficiently use fatty acid β‐oxidation (FAO) as a fuel source.^40^, ^41^ Deranged FAO in PTC leads to tubular cell injury, fibrosis, lipid accumulation and impaired energy production.^42^ We found that PGE_2_ treatment preserves lipid metabolism homeostasis after kidney injury. Furthermore, PGE_2_‐activated Sox9^+^ cells participated in intercellular communication by increasing Vegfa secretion from Sox9^+^ stem cells. Vegfa is known to maintain adipose tissue metabolism and increase energy expenditure.^43^, ^44^ Vegfa upregulates Cpt2, a key FAO enzyme associated with fatty acid oxidation and mitochondrial function,^28^, ^43^ and regulates mitochondrial biogenesis by stimulating the expression of a cluster of nuclear‐encoded mitochondrial genes.^43^, ^45^ We found that PGE_2_ treatment restored the expression of Cpt2 in PTCs. Restoration of mitochondrial β‐oxidation and/or promotion of mitochondrial biosynthesis in PTCs may be more effective in promoting renal function repair than resisting a single downstream event.^28^ This finding indicates that activated Sox9^+^ cells can regulate the restoration of PTC metabolic homeostasis via the secretion of Vegfa.

In exploring the regulatory mechanisms of differentiation, our research focuses on the influence of PGE_2_ in moderating the population reduction of fibroblasts originating from the proliferation and differentiation of Sox9^+^ cells, ultimately facilitating a decrease in renal fibrosis. The specific involvement of PGE_2_ in fibrosis via the modulation of fibroblasts from alternative origins requires further investigation. Recent studies showed that Sox9 long‐term activation has been described as a sign of maladaptive repair.^46^ In our previous PGE_2_ release study, we revealed that the concentration of PGE_2_ in the kidney returns to almost baseline level by Day 14, which excludes the long‐term Sox9 cell activation and avoids maladaptive fibrosis.^12^

In summary, we used a combination of scRNA‐seq and functional assays to identify the role of Sox9^+^ cells in renal regenerating processes in response to PGE_2_ after AKI. Through the dissection of cell differentiation dynamics, we deciphered different populations of renal tubule cells that show dynamic gene expression and provided Sox9^+^ lineage reconstruction with phenotypic information in the kidney after AKI.

## AUTHOR CONTRIBUTIONS

Z.L., X.C., X.M.C. and S.C. conceived and designed the experiments and revised the manuscript. S.C., Y.L. and H.H. performed the experiments. H.T., Y.P. and S.C. analysed the data. Z.C.H. and Z.H. provided technical support. S.C., Y.L. and Z.L. wrote the manuscript, and all authors approved the final version of this manuscript. S.C. and Y.L. contributed equally to this work.

## CONFLICT OF INTEREST STATEMENT

The authors declare no conflicts of interest.

## Supporting information


**Data S1.** Tables.

**Data S2.** Supporting Information.

## Data Availability

The data that support the findings of this study are available from the corresponding author upon reasonable request.

## References

[1] Zhang W , Gao C , Tsilosani A , Samarakoon R , Plews R , Higgins PJ . Potential renal stem/progenitor cells identified by in vivo lineage tracing. Am J Physiol Renal Physiol. 2022;322(4):F379‐F391. 35100814 10.1152/ajprenal.00326.2021 PMC8934668

[2] Wagner DE , Klein AM . Lineage tracing meets single‐cell omics: opportunities and challenges. Nat Rev Genet. 2020;21(7):410‐427. 32235876 10.1038/s41576-020-0223-2 PMC7307462

[3] Kester L , van Oudenaarden A . Single‐cell transcriptomics meets lineage tracing. Cell Stem Cell. 2018;23(2):166‐179. 29754780 10.1016/j.stem.2018.04.014

[4] Zhang K , Li R , Chen X , et al. Renal endothelial cell‐targeted extracellular vesicles protect the kidney from ischemic injury. Adv Sci (Weinh). 2023;10(3):e2204626. 36416304 10.1002/advs.202204626 PMC9875634

[5] Huang H , Qian M , Liu Y , et al. Genetically engineered mesenchymal stem cells as a nitric oxide reservoir for acute kidney injury therapy. eLife. 2023;12:e84820. 37695201 10.7554/eLife.84820 PMC10541176

[6] FitzSimons M , Beauchemin M , Smith AM , et al. Cardiac injury modulates critical components of prostaglandin E(2) signaling during zebrafish heart regeneration. Sci Rep. 2020;10(1):3095. 32080283 10.1038/s41598-020-59868-6 PMC7033201

[7] Miyoshi H , VanDussen KL , Malvin NP , et al. Prostaglandin E2 promotes intestinal repair through an adaptive cellular response of the epithelium. EMBO J. 2017;36(1):5‐24. 27797821 10.15252/embj.201694660 PMC5210160

[8] Liu Y , Ren HZ , Wang JL , et al. Prostaglandin E‐2 secreted by mesenchymal stem cells protects against acute liver failure via enhancing hepatocyte proliferation. FASEB J. 2019;33(2):2514‐2525. 30260707 10.1096/fj.201801349RR

[9] Cheng H , Huang H , Guo Z , Chang Y , Li Z . Role of prostaglandin E2 in tissue repair and regeneration. Theranostics. 2021;11(18):8836‐8854. 34522214 10.7150/thno.63396 PMC8419039

[10] Reginensi A , Clarkson M , Neirijnck Y , et al. SOX9 controls epithelial branching by activating RET effector genes during kidney development. Hum Mol Genet. 2011;20(6):1143‐1153. 21212101 10.1093/hmg/ddq558 PMC3809456

[11] Kumar S , Liu J , Pang P , et al. Sox9 activation highlights a cellular pathway of renal repair in the acutely injured mammalian kidney. Cell Rep. 2015;12(8):1325‐1338. 26279573 10.1016/j.celrep.2015.07.034

[12] Chen S , Huang H , Liu Y , et al. Renal subcapsular delivery of PGE2 promotes kidney repair by activating endogenous Sox9(+) stem cells. iScience. 2021;24(11):103243. 34746706 10.1016/j.isci.2021.103243 PMC8554536

[13] Zhang K , Chen S , Sun H , et al. In vivo two‐photon microscopy reveals the contribution of Sox9+ cell to kidney regeneration in a mouse model with extracellular vesicle treatment. J Biol Chem. 2020;295(34):12203‐12213. 32641493 10.1074/jbc.RA120.012732 PMC7443503

[14] Huang H , Chen S , Cheng H , et al. The sustained PGE(2) release matrix improves neovascularization and skeletal muscle regeneration in a hindlimb ischemia model. J Nanobiotechnol. 2022;20(1):95. 10.1186/s12951-022-01301-3 PMC8867652 35209908

[15] Satija R , Farrell JA , Gennert D , Schier AF , Regev A . Spatial reconstruction of single‐cell gene expression data. Nat Biotechnol. 2015;33(5):495‐502. 25867923 10.1038/nbt.3192 PMC4430369

[16] Aibar S , Gonzalez‐Blas CB , Moerman T , et al. SCENIC: single‐cell regulatory network inference and clustering. Nat Methods. 2017;14(11):1083‐1086. 28991892 10.1038/nmeth.4463 PMC5937676

[17] Luo WJ , Brouwer C . Pathview: an R/Bioconductor package for pathway‐based data integration and visualization. Bioinformatics. 2013;29(14):1830‐1831. 23740750 10.1093/bioinformatics/btt285 PMC3702256

[18] Chen J , Crawford R , Chen C , Xiao Y . The key regulatory roles of the PI3K/Akt signaling pathway in the functionalities of mesenchymal stem cells and applications in tissue regeneration. Tissue Eng Part B Rev. 2013;19(6):516‐528. 23651329 10.1089/ten.TEB.2012.0672

[19] Meng D , Frank AR , Jewell JL . mTOR signaling in stem and progenitor cells. Development. 2018;145(1):dev152595. 29311260 10.1242/dev.152595 PMC5825873

[20] Garcia‐Prat L , Perdiguero E , Alonso‐Martin S , et al. FoxO maintains a genuine muscle stem‐cell quiescent state until geriatric age. Nat Cell Biol. 2020;22(11):1307‐1318. 33106654 10.1038/s41556-020-00593-7

[21] Chan YH , Lee YC , Hung CY , Yang PJ , Lai PC , Feng SW . Three‐dimensional spheroid culture enhances multipotent differentiation and stemness capacities of human dental pulp‐derived mesenchymal stem cells by modulating MAPK and NF‐kB signaling pathways. Stem Cell Rev Rep. 2021;17(5):1810‐1826. 33893620 10.1007/s12015-021-10172-4

[22] Bergen V , Lange M , Peidli S , Wolf FA , Theis FJ . Generalizing RNA velocity to transient cell states through dynamical modeling. Nat Biotechnol. 2020;38(12):1408‐1414. 32747759 10.1038/s41587-020-0591-3

[23] Trapnell C , Cacchiarelli D , Grimsby J , et al. The dynamics and regulators of cell fate decisions are revealed by pseudotemporal ordering of single cells. Nat Biotechnol. 2014;32(4):381‐386. 24658644 10.1038/nbt.2859 PMC4122333

[24] Kriz W , Kaissling B , Le Hir M . Epithelial‐mesenchymal transition (EMT) in kidney fibrosis: fact or fantasy? J Clin Invest. 2011;121(2):468‐474. 21370523 10.1172/JCI44595 PMC3026733

[25] Rudnicki M , Perco P , Enrich J , et al. Hypoxia response and VEGF‐A expression in human proximal tubular epithelial cells in stable and progressive renal disease. Lab Invest. 2009;89(3):337‐346. 19139726 10.1038/labinvest.2008.158

[26] Yoshida GJ . Emerging roles of Myc in stem cell biology and novel tumor therapies. J Exp Clin Cancer Res. 2018;37(1):173. 30053872 10.1186/s13046-018-0835-y PMC6062976

[27] Seruggia D , Oti M , Tripathi P , et al. TAF5L and TAF6L maintain self‐renewal of embryonic stem cells via the MYC regulatory network. Mol Cell. 2019;74(6):1148‐1163.e7. 31005419 10.1016/j.molcel.2019.03.025 PMC6671628

[28] Peng JC , Wu T , Wu X , et al. Development of mortality prediction model in the elderly hospitalized AKI patients. Sci Rep. 2021;11(1):15157. 34312443 10.1038/s41598-021-94271-9 PMC8313696

[29] Palikaras K , Lionaki E , Tavernarakis N . Balancing mitochondrial biogenesis and mitophagy to maintain energy metabolism homeostasis. Cell Death Differ. 2015;22(9):1399‐1401. 26256515 10.1038/cdd.2015.86 PMC4532782

[30] Shang D , Sun D , Shi C , et al. Activation of epidermal growth factor receptor signaling mediates cellular senescence induced by certain pro‐inflammatory cytokines. Aging Cell. 2020;19(5):e13145. 32323422 10.1111/acel.13145 PMC7253070

[31] Doi K , Noiri E , Fujita T . Role of vascular endothelial growth factor in kidney disease. Curr Vasc Pharmacol. 2010;8(1):122‐128. 19485913 10.2174/157016110790226606

[32] Villegas G , Lange‐Sperandio B , Tufro A . Autocrine and paracrine functions of vascular endothelial growth factor (VEGF) in renal tubular epithelial cells. Kidney Int. 2005;67(2):449‐457. 15673292 10.1111/j.1523-1755.2005.67101.x

[33] Gao L , Zhong X , Jin J , Li J , Meng XM . Potential targeted therapy and diagnosis based on novel insight into growth factors, receptors, and downstream effectors in acute kidney injury and acute kidney injury‐chronic kidney disease progression. Signal Transduct Target Ther. 2020;5(1):9. 32296020 10.1038/s41392-020-0106-1 PMC7018831

[34] Li M , Zheng H , Han Y , et al. LncRNA Snhg1‐driven self‐reinforcing regulatory network promoted cardiac regeneration and repair after myocardial infarction. Theranostics. 2021;11(19):9397‐9414. 34646377 10.7150/thno.57037 PMC8490501

[35] Tang SW , Chang WH , Su YC , et al. MYC pathway is activated in clear cell renal cell carcinoma and essential for proliferation of clear cell renal cell carcinoma cells. Cancer Lett. 2009;273(1):35‐43. 18809243 10.1016/j.canlet.2008.07.038

[36] Clevers H , Loh KM , Nusse R . An integral program for tissue renewal and regeneration: Wnt signaling and stem cell control. Science. 2014;346(6205):1248012. 25278615 10.1126/science.1248012

[37] Varlakhanova NV , Cotterman RF , deVries WN , et al. myc maintains embryonic stem cell pluripotency and self‐renewal. Differentiation. 2010;80(1):9‐19. 20537458 10.1016/j.diff.2010.05.001 PMC2916696

[38] Sato T , Okumura F , Ariga T , Hatakeyama S . TRIM6 interacts with Myc and maintains the pluripotency of mouse embryonic stem cells. J Cell Sci. 2012;125(Pt 6):1544‐1555. 22328504 10.1242/jcs.095273

[39] Chang‐Panesso M , Humphreys BD . Cellular plasticity in kidney injury and repair. Nat Rev Nephrol. 2017;13(1):39‐46. 27890924 10.1038/nrneph.2016.169

[40] Li M , Li CM , Ye ZC , et al. Sirt3 modulates fatty acid oxidation and attenuates cisplatin‐induced AKI in mice. J Cell Mol Med. 2020;24(9):5109‐5121. 32281286 10.1111/jcmm.15148 PMC7205836

[41] Bataille A , Galichon P , Chelghoum N , et al. Increased fatty acid oxidation in differentiated proximal tubular cells surviving a reversible episode of acute kidney injury. Cell Physiol Biochem. 2018;47(4):1338‐1351. 29929186 10.1159/000490819

[42] Emma F , Montini G , Parikh SM , Salviati L . Mitochondrial dysfunction in inherited renal disease and acute kidney injury. Nat Rev Nephrol. 2016;12(5):267‐280. 26804019 10.1038/nrneph.2015.214 PMC5469549

[43] Zhao Y , Li X , Yang L , et al. Transient overexpression of vascular endothelial growth factor a in adipose tissue promotes energy expenditure via activation of the sympathetic nervous system. Mol Cell Biol. 2018;38(22):e00242‐18. 30126894 10.1128/MCB.00242-18 PMC6206456

[44] di Somma M , Vliora M , Grillo E , et al. Role of VEGFs in metabolic disorders. Angiogenesis. 2020;23(2):119‐130. 31853841 10.1007/s10456-019-09700-1

[45] Wright GL , Maroulakou IG , Eldridge J , et al. VEGF stimulation of mitochondrial biogenesis: requirement of AKT3 kinase. FASEB J. 2008;22(9):3264‐3275. 18524868 10.1096/fj.08-106468 PMC2518259

[46] Aggarwal S , Wang Z , Rincon Fernandez Pacheco D , et al. SOX9 switch links regeneration to fibrosis at the single‐cell level in mammalian kidneys. Science. 2024;383(6685):eadd6371. 38386758 10.1126/science.add6371 PMC11345873

