# Supplementary material for: Combined lineage tracing and scRNA‐seq reveal the activation of Sox9+ cells in renal regeneration with PGE2 treatment
Source: Cell Prolif. 2024 May 27;57(11):e13679. doi: 10.1111/cpr.13679 (PMC11533080; doi:10.1111/cpr.13679)
Supplement: Supplementary file 2 — Data S2. Supporting Information. [file CPR-57-e13679-s002.docx]

**Supporting information**

**Supplemental Methods**

**Western blot analysis**

Sox9^+^ cells isolated from Sox9-Cre^ERT2^; Rosa26^mTmG^ mice treated with tamoxifen (100 mg/kg) for 3 days were plated onto uncoated, collagen or PGE_2_ matrix (2 μM)-coated 6-well cells and treated with wortmannin (1 μM) or DMSO as control for 48 h. The treated cells were lysed in RIPA buffer (Solarbio, Shanghai, China) with a proteinase inhibitor cocktail (Sigma-Aldrich) for 20 min on ice. For tissue protein analysis, renal capsules, and matrix were removed from mouse kidney tissue with COL-PGE_2_ treatment for 28 days, cut into a 1.5 mL EP tube supplemented with a proteinase inhibitor cocktail, and transferred to a homogenizer. The tissue homogenates were lysed on ice with RIPA buffer for 20 min after total protein quantification using a BCA Protein Assay Kit (Thermo Scientific). Total proteins were diluted in 4× SDS-PAGE loading buffer and boiled for 5 min. The harvested proteins were separated by 10% SDS‒PAGE and transferred to polyvinylidene fluoride membranes (PVDF; Millipore, Darmstadt, Germany). Primary antibodies included Gapdh (60004-1-Ig, 1:1000, Proteintech Group, Wuhan, China), Pi3k (sc-1637, 1:500, Santa Cruz Biotechnology, USA), Phospho-Pi3k (17366, 1:000, Cell Signaling Technology, USA), Akt (sc-5298, 1:500, Santa Cruz Biotechnology, USA), Phospho-Akt (sc-377556, 1:500, Santa Cruz Biotechnology, USA), Myc (sc-40, 1:500, Santa Cruz Biotechnology) and Vegfa (ab46154, 1:000, Abcam, UK),Snail (3879, 1:000, Cell Signaling Technology, USA), Slug(9585, 1:000, Cell Signaling Technology, USA), Vimentin(46173, 1:000, Cell Signaling Technology, USA), Col1a1(81375, 1:000, Cell Signaling Technology, USA), Pcna(13110, 1:000, Cell Signaling Technology, USA), Cpt2 (sc-377294, 1:500, Santa Cruz Biotechnology, USA), Il-6(12912, 1:000, Cell Signaling Technology, USA), P16(sc-1661, 1:500, Santa Cruz Biotechnology, USA), P21(sc-6246, 1:500, Santa Cruz Biotechnology, USA).

**Histological analysis of renal tissues**

Mice were sacrificed and kidneys were collected on days 3, 7, 14, and 28 after PGE_2_ treatment. Kidney samples were fixed with 4% paraformaldehyde. Masson trichrome staining and oil Red O staining were performed. For immunofluorescent staining, cryosections were incubated with primary antibodies against Vegfa (ab46154, 1:200, Abcam, UK), Ki-67 (12075, 1:200, Cell Signaling Technology, USA), Myc (sc-40, 1:50, Santa Cruz Biotechnology, USA), Sox9 (1:200 ab185966, Abcam, UK), Sox9(sc-166505, 1:50, Santa Cruz Biotechnology, USA), F4/80 (ab16911, 1:200, Abcam, UK) and Cpt2 (sc-377294, 1:50, Santa Cruz Biotechnology, USA), Kim-1(ab47635, 1:200, Abcam, UK). The sections were incubated with appropriate fluorescently labeled secondary antibodies (Life Technologies, Carlsbad, CA). A mounting solution containing 4’-6-diamidino-2-phenylindole (DAPI; C1002, 1:10000, Beyotime Biotechnology, China) was used to mount the sections.

**Single-cell RNA sequencing and data analysis**

For single-cell RNA-sequencing (scRNA-seq) of two pooled EGFP-positive cell samples obtained from the control and PGE_2_ matrix treatment groups of C57BL/6 Sox9-Cre^ERT2^; Rosa26^mTmG^ transgenic mice after AKI, Chromium™ Single Cell 3′ Reagent Kit v3 chemistry (10× Genomics) was used and a standard protocol was followed. The library was generated and sequenced on a NovaSeq 6000 platform (Illumina) with a paired-end 150 bp (PE 150) sequencing strategy. Raw single-cell RNA sequence data were processed using Cell Ranger (version 4.0.0), resulting in the two datasets of the control and PGE_2_ matrix. After aggregation of the samples, Seurat (V3.2.2, https://satijalab.org/seurat/), Monocle2 (V2.18.0, http://cole-trapnell-lab.github.io/monocle-release/), iTalk (V0.1.0, https://github.com/Coolgenome/iTALK), Senic (V1.2.2, https://scenic.aertslab.org/), and RNA scvelo (V0.2.4, https://scvelo.readthedocs.io/) packages were used for downstream analysis, including cell clustering and identification of DEGs.

**Assignment of cell identity in each cluster**

For scRNA-seq data from control and PGE_2_ treatment mice, we assigned cell types/identities to each cell using differential expression signatures derived from steady state data. Specifically, we collected up to 30 top genes most differentially expressed in each of the 8 original clusters of the homeostasis data set as signatures. For each candidate cell, we computed its signature scores for each of the 30 signatures using FindMarkers of Seurat package. Each signature score was computed against a set of background genes of randomly selected genes. A cell was assigned to the cell-type/cluster with the best signature score. When assigning the sub-clustersubcluster identity, we further scored against signature genes of sub-clusters that consisted of up to 30 most differentially expressed sub-cluster genes.

**Differential expression in each cluster**

Separately for each cluster, we computed genes that were differentially expressed between the PGE_2_ treatment group and the control in two ways. First, we used Bonferroni corrected Wilcoxon Rank-Sum Test (FindMarkers of Seurat package) to discover differentially expressed genes between conditions. Second, for each cluster, we computed the average gene expression of cells in every replicate, |log_2_FC|>0.5, FDR < 0.05. Then, we used these values in a t-test to assess differences between the PGE_2_ treatment group and the control conditions, |log_2_FC| > 0.25, p value<0.05. This approach mimics bulk RNA-seq measurements.

For the analysis of differentially expressed genes in each cluster between two groups, we used gene sets Signaling pathways regulating pluripotency of stem cells", "Pi3k-Akt signaling pathway" from KEGG, "CELL_PROLIFERATION_GO_0008283", and "GOBP_REGENERATION" from GSEA. Human gene names were first converted to mouse gene names before analysis. Gene set enrichment analysis of KEGG pathway was analyzed using Metascape bioinformatics resources.

**Inferring gene-regulatory networks**

To identify regulators that are active in different populations, the SCENIC ^1^ algorithm was used to infer the activity of the regulon in each cell state. The analysis pipeline was performed following the instructions of developers (V1.2.2 https://github.com/aertslab/SCENIC). The regulons were clustered with a supervised analysis to obtain lists of the regulons with differential activity amongst each cell type. In short, the expression matrix was first extracted from Seurat and further converted to the format required by SCENIC (rows represent genes, columns represent cells). We then used the GENIE3 R package to identify co-expressed gene modules and infer potential TF targets for each module from the expression matrix. Subsequently, RcisTarget was used to perform cis-regulatory motif analysis, two motifs were scanned into the TFs database (mm9-500bp-upstream-7species.mc9nr and mm9-tss-centered-10kb-7species.mc9nr; https://resources.aertslab.org/cistarget/) and retain modules with significant motif enrichment; these modules were then termed as regulons according to the SCENIC pipeline.

**Cell trajectory and pseudo-time analysis**

Monocle analysis is an algorithm that arranges cells along a bioinformatic trajectory that minimizes differences in gene expression between neighboring cells^2-5^. Monocle 2(v2.18.0) was used for the pseudotemporal ordering of the combined dataset and the individual (control and PGE_2_ group) dataset. Monocle 2 used a DDR-Tree (Discriminative DRTree) for dimensionality reduction and tree construction. In short, an aggregated gene expression matrix was constructed. Differential expression genes were identified under different developmental conditions using the differential gene test function of Monocle 2. Monocle 2 was used to construct pseudo-time trajectories using the first 2,000 genes with the lowest q value. Based on its position along the trajectory, every cell was assigned a pseudo-time value. The actual time of each cell identifies which cell states in "orderCells" were at the beginning of pseudo-time. "DDRTree" and "UMAP" were used to reduce dimensional space. Using “plot_complex_cell_trajectory”, “plot_pseudotime_heatmap” and “plot_genes_branched_heatmap” of Monocle 2 plotted minimum spanning trees of cells and heat maps along pseudotime, respectively.

**Gene set enrichment analysis**

We used Gene Set Enrichment Analysis (GSEA, V4.1.0) with MSigDB (v7.5) gene sets (“HP_NEPHROTIC_SYNDROME”, “HP_NEPHRITIS”, “HALLMARK_OF_EPITHELIAL_MESENCHYMAL_TRANSITION”, “REACTOME_METABOLISM_OF_LIPIDS”, “HALLMARK_FATTY_ACID_METABOLISM”, “GOBP_OXIDATIVE_PHOSPHORYLATION”, “REACTOME_MITOCHONDRIAL_BIOGENESIS”,

“WP_EMBRYONIC_STEM_CELL_PLURIPOTENCY_PATHWAYS” and “GOBP_AUTOPHAGY_OF_MITOCHONDRION”), KEGG gene set (“Signaling pathways regulating pluripotency of stem cells”) and “Kidney Panel” to identify pathways and cellular states that have induced or repressed expression. Specifically, we used the pre-ranked analysis mode, with gene transcripts ranked according to differential expression analysis results (Wilcoxon Runk-Sum Test) of comparing the PGE_2_ treatment group and control conditions in each cluster.

**RNA velocity analysis**

RNA velocity analysis was performed using the dynamical model of the scVelo package (v0.2.4) ^6^. The dynamical model of the scVelo package was used for RNA velocity analysis, leverage ratios of spliced versus unspliced RNA molecules to predict future states of cells. We used the same genome annotation files used for CellRanger for alignment, and the GRCh38 repeat mask files were downloaded from the UCSC genome browser. Cells previously passed QC and subtyped in previous gene expression analysis were extracted from the velocity output. In order to find genes that have different regulatory transcriptional dynamics compared with all other clusters, the Welch t-test with overestimated variance was applied using SC.tl. rank_velocity_genes function. The genes were sequenced according to the likelihood derived from the dynamic model grouped by treatment.

**Intercellular crosstalk between cell types**

To explore intercellular crosstalk among major different cell types, we used the R package iTALK^7^ (V0.1.0, https://github.com/Coolgenome/iTALK) to identify significant intercellular ligand-receptor pairs among cell types. In summary, the top 50% of most upexpressed genes were included in the analysis. The up-regulated genes of each cell type were matched with the 2,648 unique receptor-ligand pairs recorded in the iTALK package (including growth factors, cytokines, checkpoints, and other factors). Significant cellular interactions were identified using iTALK.

**Pathway Analysis**

Pathview is an online package set of tools for path-based data integration and visualization, accessible at pathview.uncc.edu. It maps and renders differentially expressed genes (DEGs) relative expression between PTC-2 and other clusters in combined datasets on relevant pathway graphs. Pathway graphs are automatically downloaded and mapped to the pathways. This tool is used for pathway and gene set enrichment analysis^8,9^**.**

**Intravital two-photon imaging in mice**

An AIW was implanted in the abdomen of the mouse, fixed with an adapter and maintained under anesthesia by injecting avertin. The objective lens was 25× and immersed in water on the AIW window in the abdomen of the mouse. Two-photon excitation was performed at a wavelength of 835 nm (10% laser transmissivity), and emission was collected at 495–540 nm (EGFP) and 575–630 nm (td Tomato). Scanning was advanced with Z steps of 5 μm and zoom under the 25× objective lens (580 μm × 580 μm single scanning area) and an 800 × 800-pixel size.

**Cell cycle analysis**

We used “CellCycleScoring” program in Seurat R package to calculate the average expression of cell cycle-related gene sets, cell cycle analysis was performed using the previously reported core gene set, including 43 S genes(including Mcm5, Pcna, Tyms, Fen1, Mcm7, Mcm4, Rrm1, Ung, Gins2, Mcm6, Cdca7, Dtl, Prim1, Uhrf1, Cenpu, Hells, Rfc2, Polr1b, Nasp, Rad51ap1, Gmnn, Wdr76, Slbp, Ccne2, Ubr7, Pold3, Msh2, Atad2, Rad51, Rrm2, Cdc45, Cdc6, Exo1, Tipin, Dscc1, Blm, Casp8ap2, Usp1, Clspn, Pola1, Chaf1b, Mrpl36, E2f8) and 54 G2/M genes( including Hmgb2, Cdk1, Nusap1, Ube2c, Birc5, Tpx2, Top2a, Ndc80, Cks2, Nuf2, Cks1b, Mki67, Tmpo, Cenpf, Tacc3, Pimreg, Smc4, Ccnb2, Ckap2l, Ckap2, Aurkb, Bub1, Kif11, Anp32e, Tubb4b, Gtse1, Kif20b, Hjurp, Cdca3, Jpt1, Cdc20, Ttk, Cdc25c, Kif2c, Rangap1, Ncapd2, Dlgap5, Cdca2, Cdca8, Ect2, Kif23, Hmmr, Aurka, Psrc1, Anln, Lbr, Ckap5, Cenpe, Ctcf, Nek2, G2e3, Gas2l3, Cbx5, Cenpa )^10,11^. We calculate the average expression of each gene set as the corresponding score and compare the S and G2/M scores of PGE_2_-treated and control kidneys in each cluster, respectively for comparing the proliferation status of cell type. If the S score < 0 and the G2 / M score < 0, the cells were determined to be at rest. Otherwise, they were considered proliferative. Furthermore, proliferating cells were named G2/M if their G2/M score > S score, while cells were designated S if their S score > G2/M score.

**References**

1. Aibar S, Gonzalez-Blas CB, Moerman T, et al. SCENIC: single-cell regulatory network inference and clustering. *Nat Methods* 2017; **14**(11): 1083-+.

2. Trapnell C, Cacchiarelli D, Grimsby J, et al. The dynamics and regulators of cell fate decisions are revealed by pseudotemporal ordering of single cells. *Nat Biotechnol* 2014; **32**(4): 381-6.

3. Qiu X, Mao Q, Tang Y, et al. Reversed graph embedding resolves complex single-cell trajectories. *Nature methods* 2017; **14**(10): 979-82.

4. Cao JY, Spielmann M, Qiu XJ, et al. The single-cell transcriptional landscape of mammalian organogenesis. *Nature* 2019; **566**(7745): 496-+.

5. Rust K, Byrnes LE, Yu KS, et al. A single-cell atlas and lineage analysis of the adult Drosophila ovary. *Nature communications* 2020; **11**(1).

6. Bergen V, Lange M, Peidli S, Wolf FA, Theis FJ. Generalizing RNA velocity to transient cell states through dynamical modeling. *Nat Biotechnol* 2020; **38**(12).

7. Wang Y, Wang R, Zhang S, et al. iTALK: an R package to characterize and illustrate intercellular communication. *BioRxiv* 2019: 507871.

8. Luo WJ, Brouwer C. Pathview: an R/Bioconductor package for pathway-based data integration and visualization. *Bioinformatics* 2013; **29**(14): 1830-1.

9. Luo WJ, Pant G, Bhavnasi YK, Blanchard SG, Brouwer C. Pathview Web: user friendly pathway visualization and data integration. *Nucleic Acids Res* 2017; **45**(W1): W501-W8.

10. Tirosh I, Izar B, Prakadan SM, et al. Dissecting the multicellular ecosystem of metastatic melanoma by single-cell RNA-seq. *Science* 2016; **352**(6282): 189-96.

11. Macosko EZ, Basu A, Satija R, et al. Highly Parallel Genome-wide Expression Profiling of Individual Cells Using Nanoliter Droplets. *Cell* 2015; **161**(5): 1202-14.

**Supplemental Figures**

**
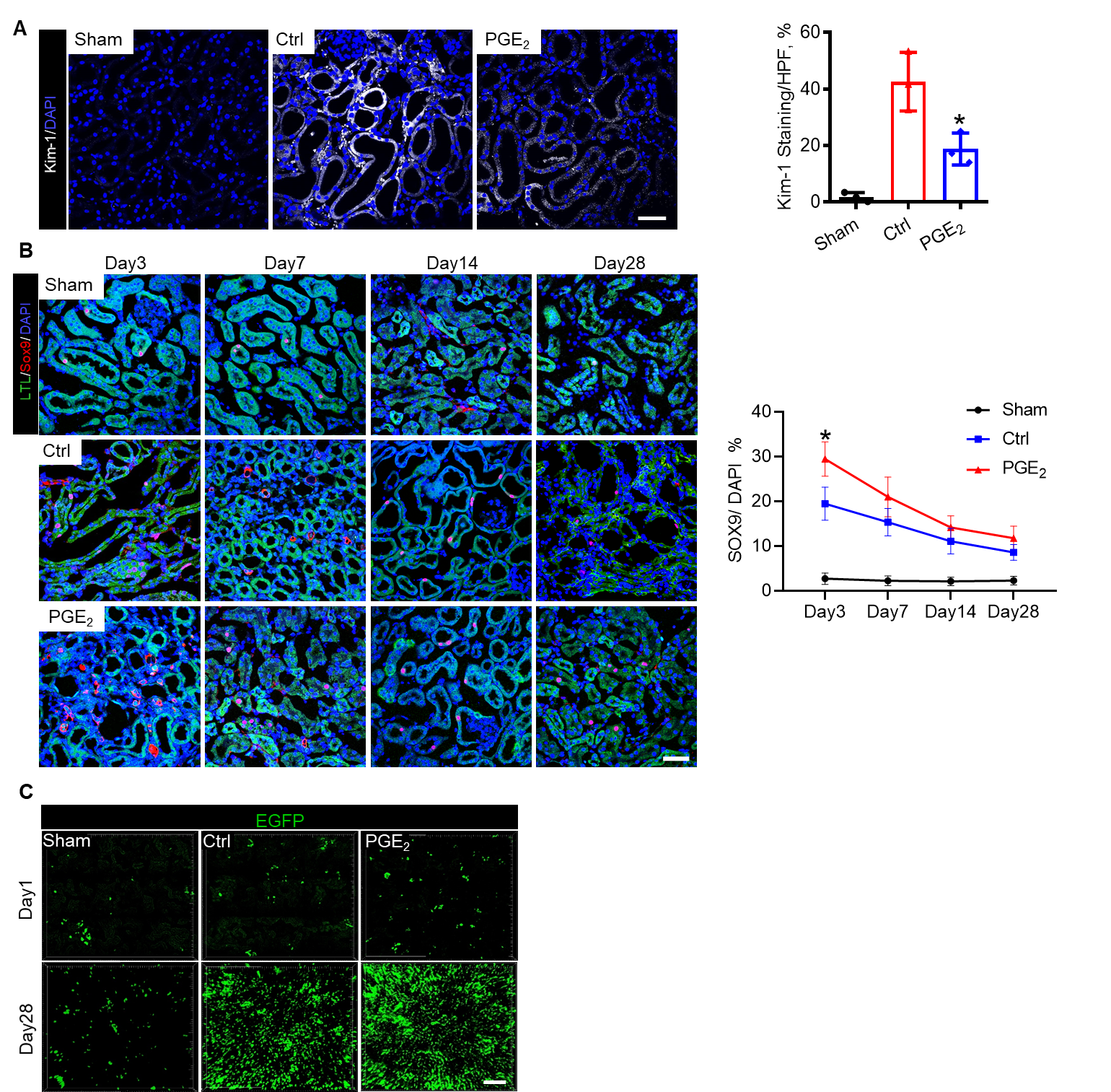
**

**Figure S1** **PGE_2_ could activate renal Sox9+ cells.** (**A**) Histological analysis of anti-Kim-1 immunostaining (gray) on day 3 post AKI. Scale bars, 50 μm. HPF, high-power field. (**B**) Representative images and quantification of Sox9 (red) immunofluorescence staining on days 3, 7, 14, and 28 after AKI. The proximal tubules were co-stained with lotus tetragonolobus lectin (LTL; green). Scale bars, 50 μm. (**C**) Representative images of the two-photon intravital tracing showed that Sox9^+^ cell derived cells (EGFP^+^ cells) expanded abundantly and formed tubular structures after PGE_2_ treatment after AKI. Treatment with PGE_2_ promoted the proliferation of Sox9-positive cells into progeny cells. Scale bar, 200 μm.


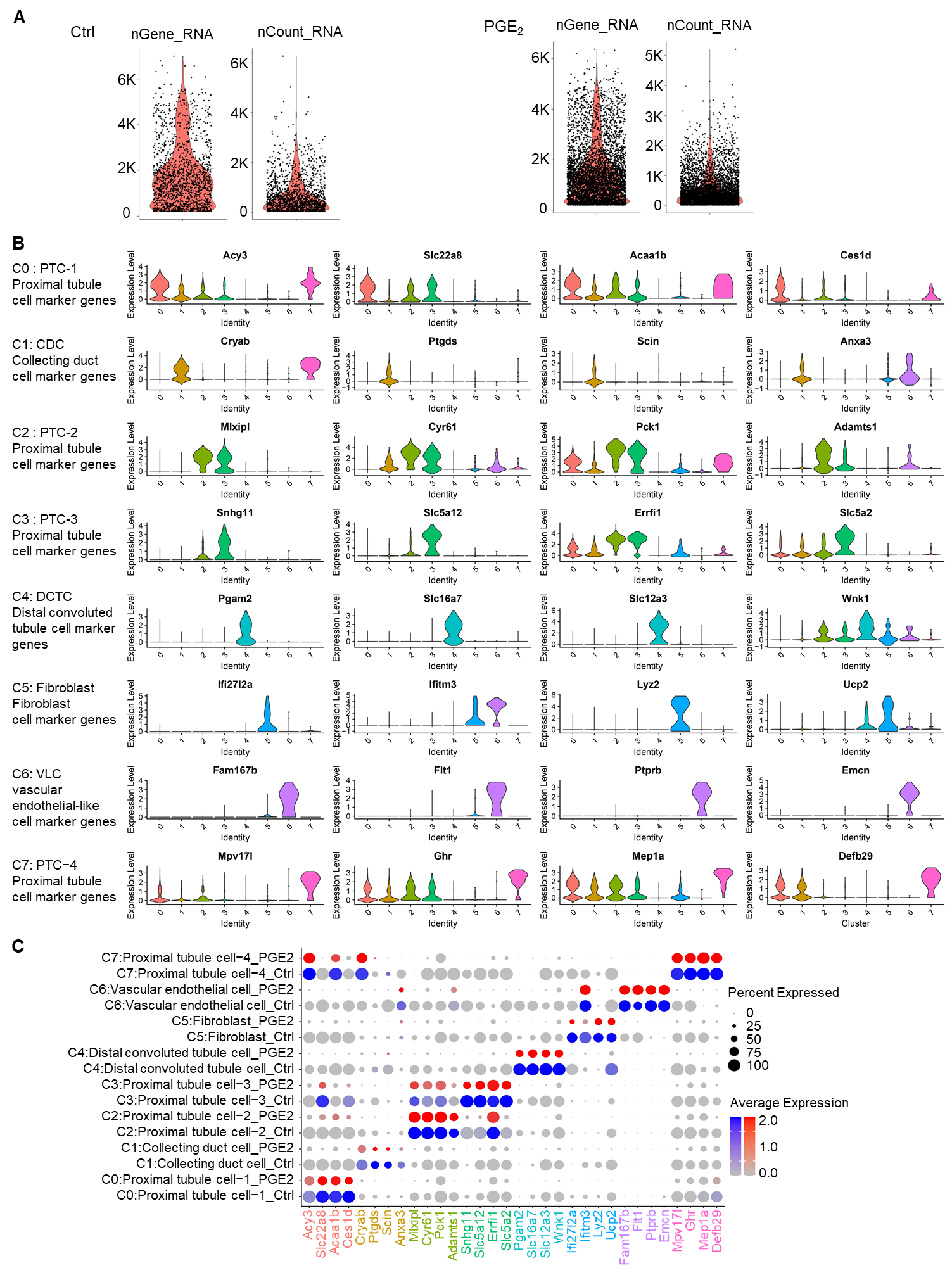


**Figure S2 The violin plots revealed the expression patterns of each cluster according to cluster-specific marker genes.** (**A**) Scatter diagram showing the gene numbers (nGene_RNA) (left) and transcript RNA molecules of single cells (nCount_RNA) (right). (**B**) Violin plot of selected markers indicating the level of expression of canonical markers within each type (e.g., Proximal tubule cell marker genes: Acy3, Slc22a8, Acaa1b, Ces1d, Mlxipl, Cyr61, Pck1, Adamts1, Snhg11, Slc5a12, Errfi1, Slc5a2, Mpv17l, Ghr, Mep1a, Defb29. Collecting duct cell marker genes: Cryab, Ptgds, Scin, Anxa3. Distal convoluted tubule cell marker genes: Pgam2, Slc16a7, Slc12a3, Wnk1. Fibroblast marker genes: Ifi27l2a, Ifitm3, Lyz2, Ucp2. Vascular endothelial-like cell marker genes: Fam167b, Flt1, Ptprb, Emcn). Violin plots are colored by cell type and the width represents the percentage of cells expressing the marker at a given expression level in Log2FC from cluster 0 to cluster 7. (**C**) The cell clusters were identified by expression of a marker specific for the kidney cell lineage. Dot plot showing the average expression (color-scaled) of selected cell marker genes in each cell cluster and different treatment groups. The dot size reflects the proportion of cells expressing the selected gene.


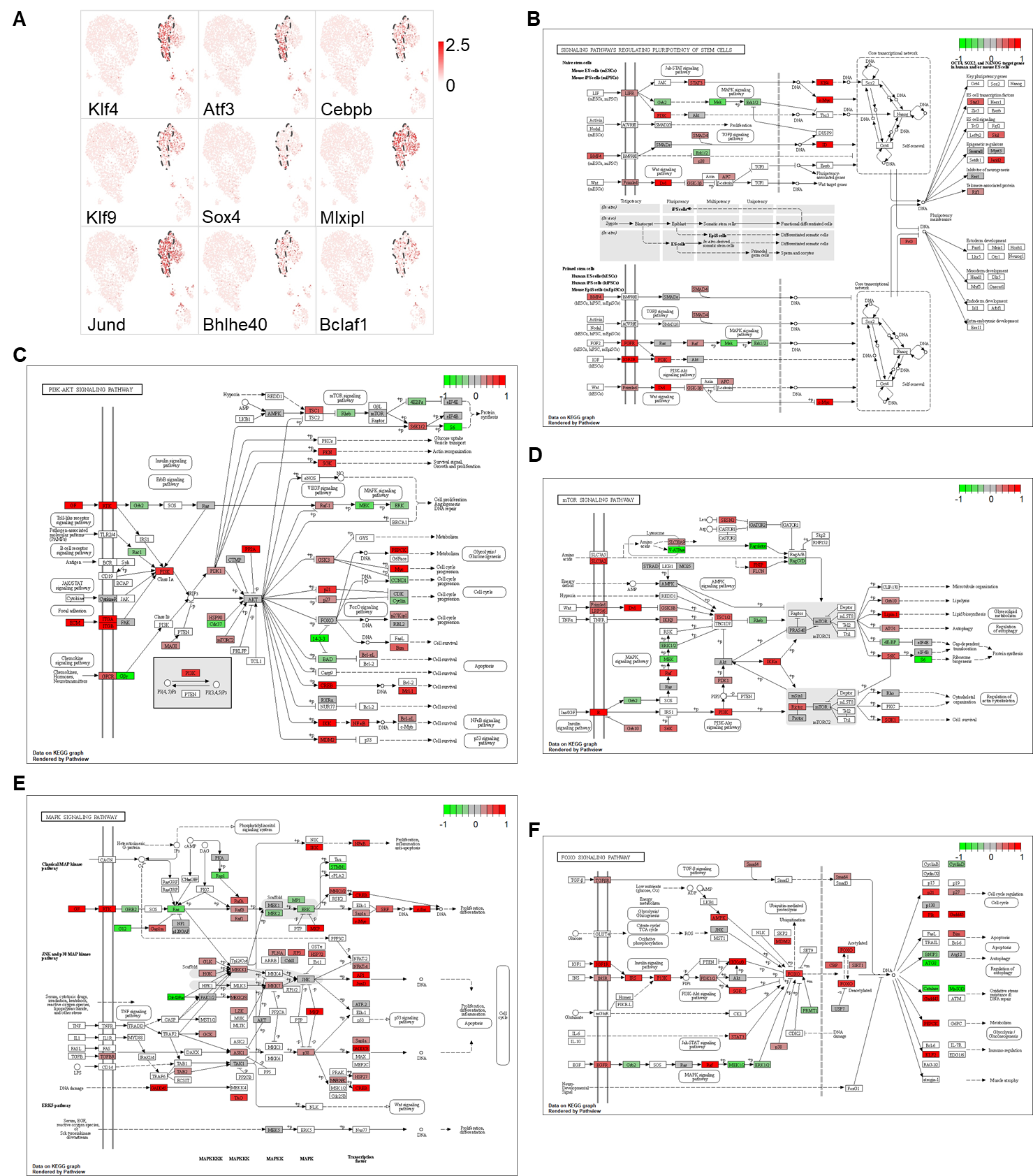


**Figure S3 Transcription factors known to be involved in cell pluripotency. (A)** Reciprocal activity of regulons related to stem cell characteristics and cell proliferation on single-cell data. The area enclosed by the black dotted lines represents PTC-2. “ Klf4, Atf3, Cebpb, Klf9, and Sox4" were related to the self-renewal and pluripotency characteristics of stem cells, and "Mlxipl, Jund, Bclaf1, and Bhlhe40" were related to cell proliferation and were mainly enriched in PTC-2. **(B-F)** Visualization of the upregulated differentially expressed genes (DEGs) in PTC-2 on the signaling pathways that regulate pluripotency of stem cells pathway, the Pi3k-Akt signaling pathway, the mTOR signaling pathway, Mapk signaling pathway and the FoxO signaling pathway. Green indicates downregulated expression, red indicates upregulated expression, and gray indicates unchanged expression vs. all other cell types.


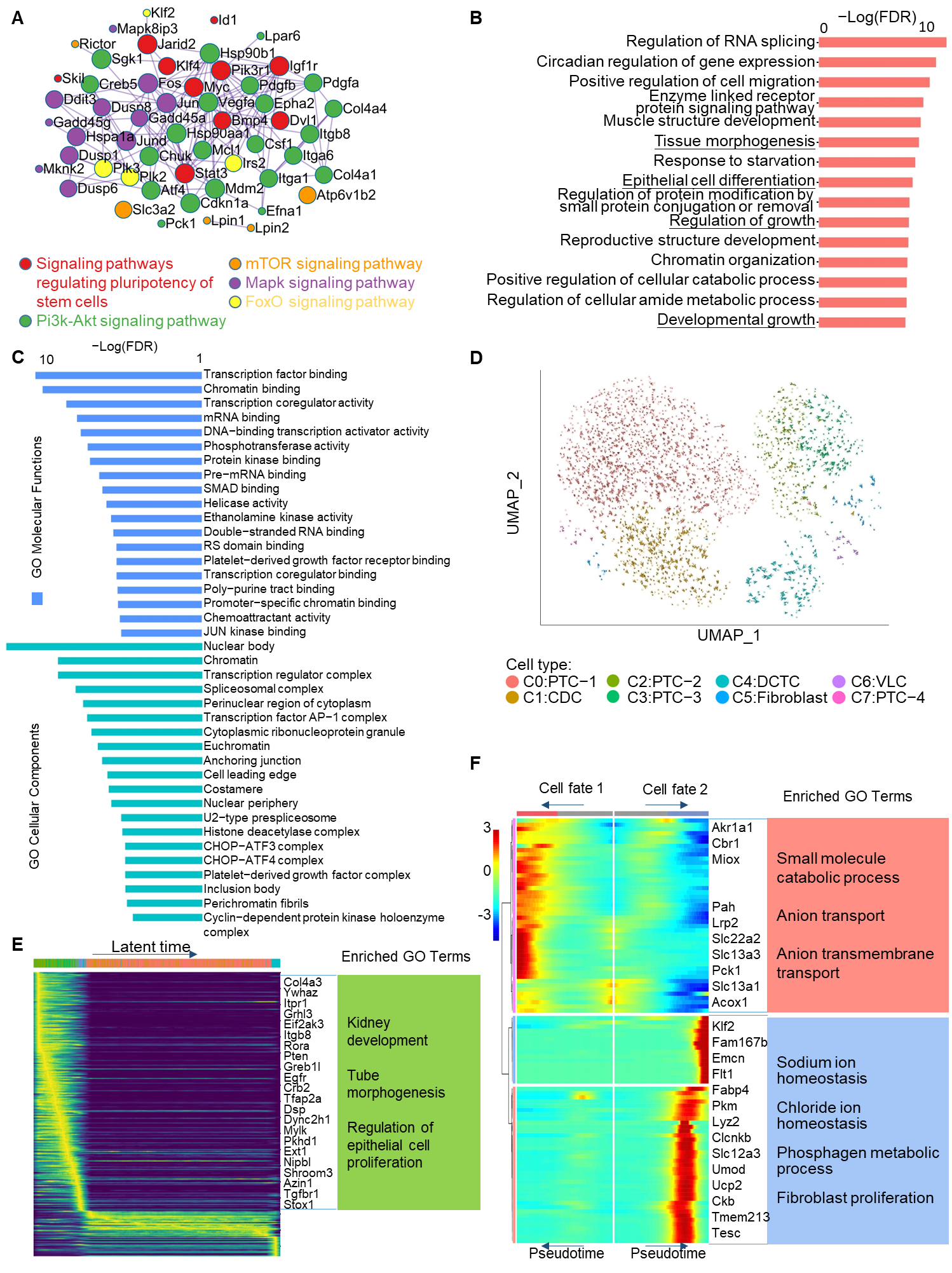


**Figure S4** **The functional status of Sox9+ cells in the PTC-2 cluster.** (**A**) Protein-protein interaction (PPI) network of the genes selected in Fig.3D. (**B**) Gene Ontology (GO) biological process enrichment analysis revealed the increased DEGs in PTC-2 for tissue morphogenesis, epithelial cell differentiation, developmental growth, and regulation of growth biological processes. log_2_FC >0.5, p value < 0.05. (**C**) GO cellular component and molecular function process enrichment analysis result of up-regulated differentially expressed genes (DEGs) in PTC-2 with log2FC >0.5, p value<0.05. The enrichment of “Cyclin−dependent protein kinase holoenzyme complex, Transcription factor binding, Transcription coregulator activity, Promoter−specific chromatin binding, Protein kinase binding” indicated that the PTC-2 cell cluster had the high proliferative state (**D**) RNA velocities were visualized on the U-MAP projection of all cells from multiple donors using Gaussian smoothing on a regular grid for each cell. (**E**) Heatmap displaying predicted scaled expression of all cell types' genes across latent time in combined datasets of PGE_2_ and control. Labeled genes were predicted to be up-regulated. Col4a3, Ext1, Tfap2a, Tgfbr1, Osr2, Dync2h1, Pkhd1, Greb1l belong to the "kidney development" gene set. Egfr, Eif2ak3, Pten, Rora, Ywhaz, Shroom3, Nipbl, Mylk, Grhl3, Itgb8, Dsp, Crb2, Itpr1, Azin1, Stox1 belong to the " tube morphogenesis" and "regulation of the epithelial cell proliferation" gene set, which were downregulated along the latent time GO terms enriched for each gene set of PTC-2 were labeled in the right panel. (**F**) Heatmap illustrating the dynamics expression of the top 100 DEGs towards two cell fates along pseudotime at the first fate branchpoint of combined datasets. In cell fate 1, proximal tubule cell markers (for example, Akr1a1, Miox, Pah, Lrp2, Slc13a3, Slc22a2, Pck1, Slc13a1, Acox1) were up-regulated along pseudo-timeline. In cell fate 2, distal convoluted tubule cell markers (for example, Ckb, Tesc, Umod, Slc12a3), collecting duct cell markers (for example, Clcnkb, Tmem213), vascular endothelial-like (for example, Fam167b, Flt1, Emcn, Fabp4) and fibroblast markers (for example, Lyz2, Ucp2) were up-regulated along pseudo-timeline.


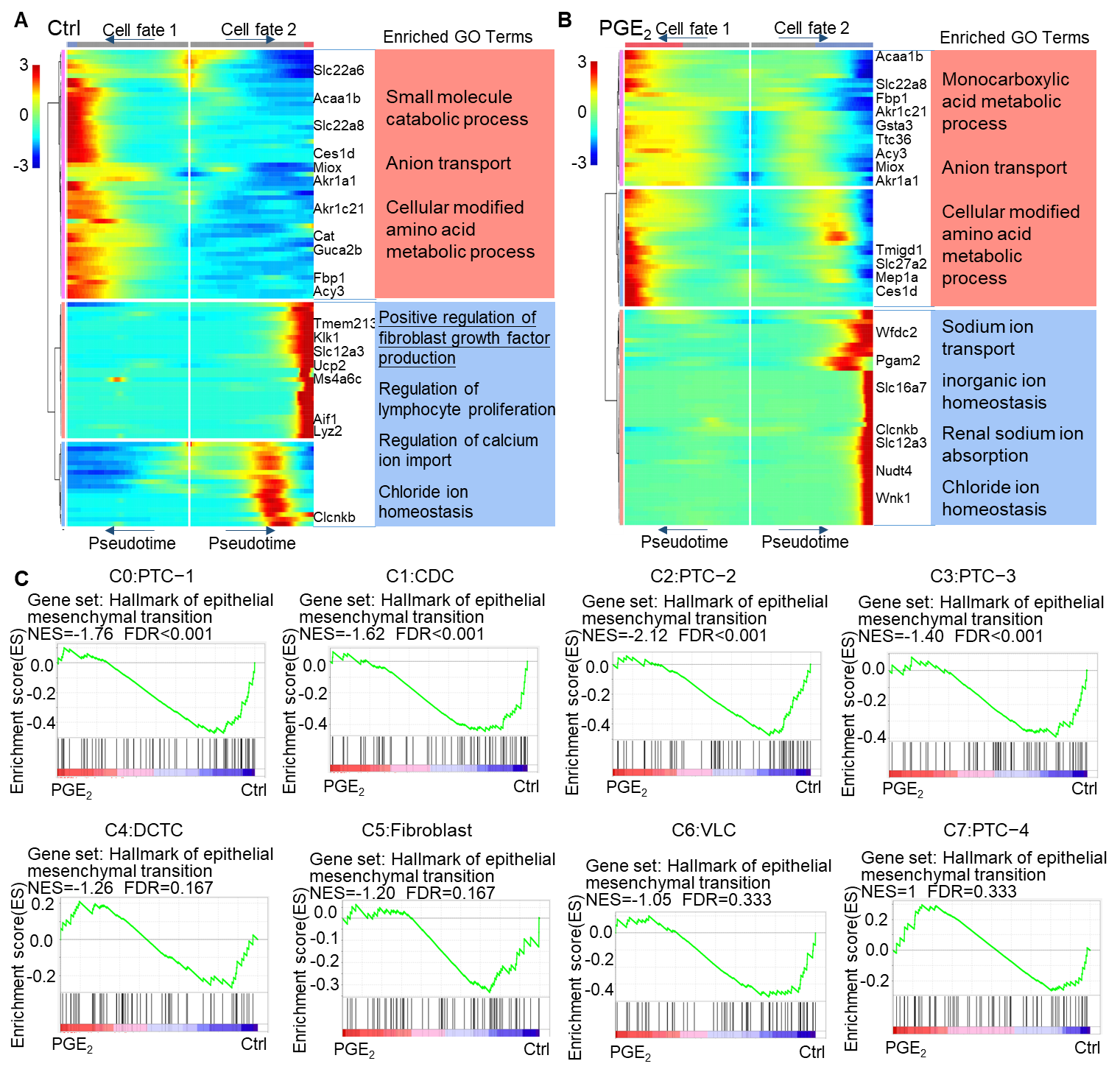


**Figure S5** **GO enrichment analysis of DEG dynamics toward cell fate.** (**A**) Heatmap illustrating the dynamic expression of the top 100 DEGs toward two cell fates along pseudotime at the first fate branchpoint of the control. In cell fate 2, distal convoluted tubule cell markers (Slc12a3, Klk1), collecting duct cell markers (for example, Clcnkb, Tmem213), and fibroblast markers (Ucp2, Ms4a6c, Lyz2) were upregulated along pseudo-time line. GO analysis, see Fig.S5A, B. (**B**) Heatmap illustrating the dynamic expression of the top 100 DEGs toward two cell fates along pseudo-time at the first fate branchpoint of the PGE_2_-treated group. GO analysis, see Fig.S5C, D. (**C**) GSEA enrichment plots from the hallmark gene dataset associated with epithelial mesenchymal transition in each cluster of the PGE_2_-treated group compared to the control.


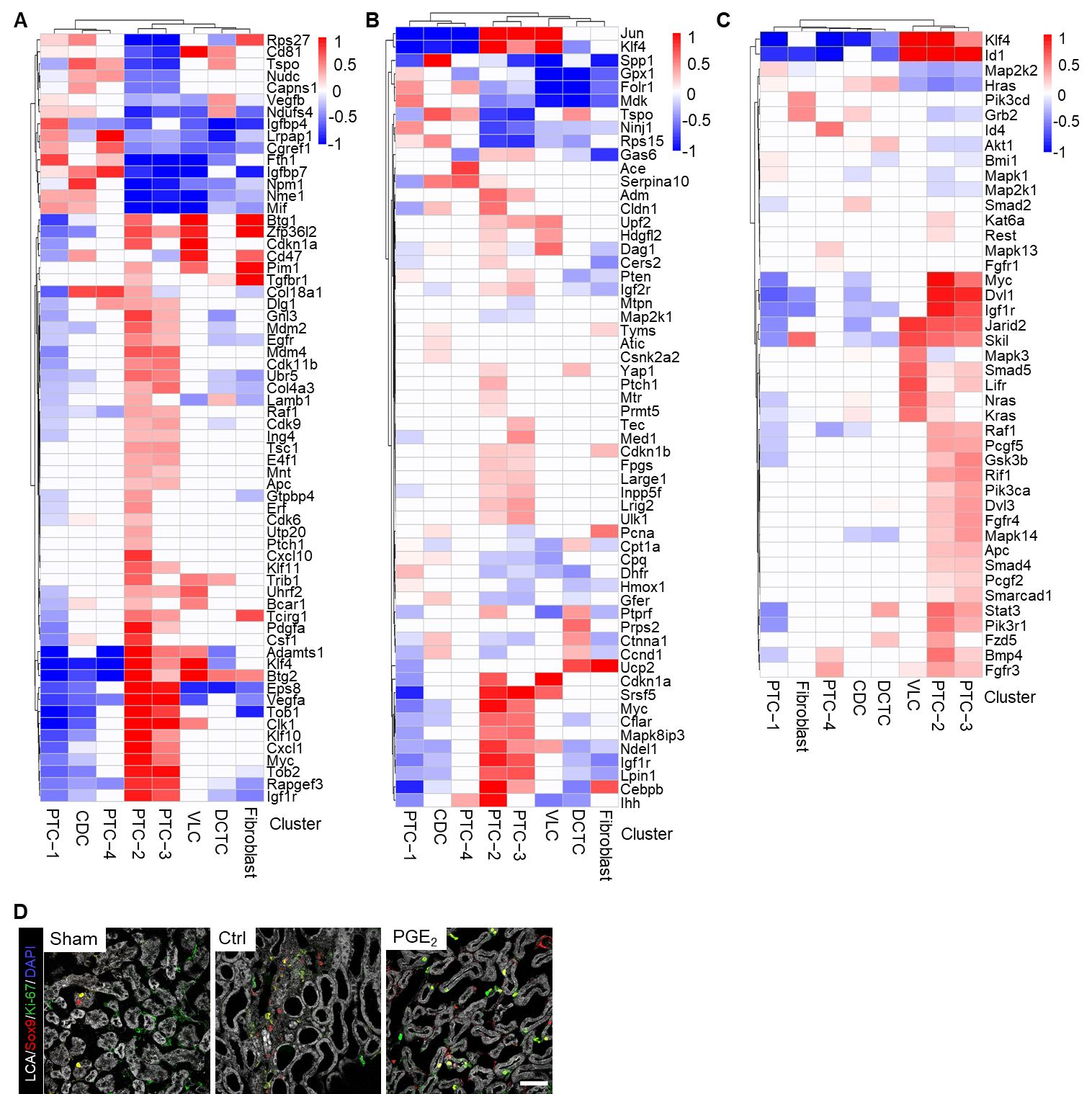


**Figure S6** **Heatmap showing the expression of DEGs enriched by functional blocks. (A-C)** Related to cell proliferation (**A**), regeneration (**B**) and stem cell pluripotency (**C**) in each cell type of the combined datasets from the control and PGE_2_ treatment groups, respectively. The color bar represents the relative gene expression, p value<0.05. Complete lists of DEGs among the clusters in the combined kidney are listed in **Table S4**. (**D**) Co-localization analysis of Sox9 and Ki67 on day 3 after AKI. Scale bar, 50 μm. The proximal tubules were co-stained with lens culinaris agglutinin (LCA; gray).


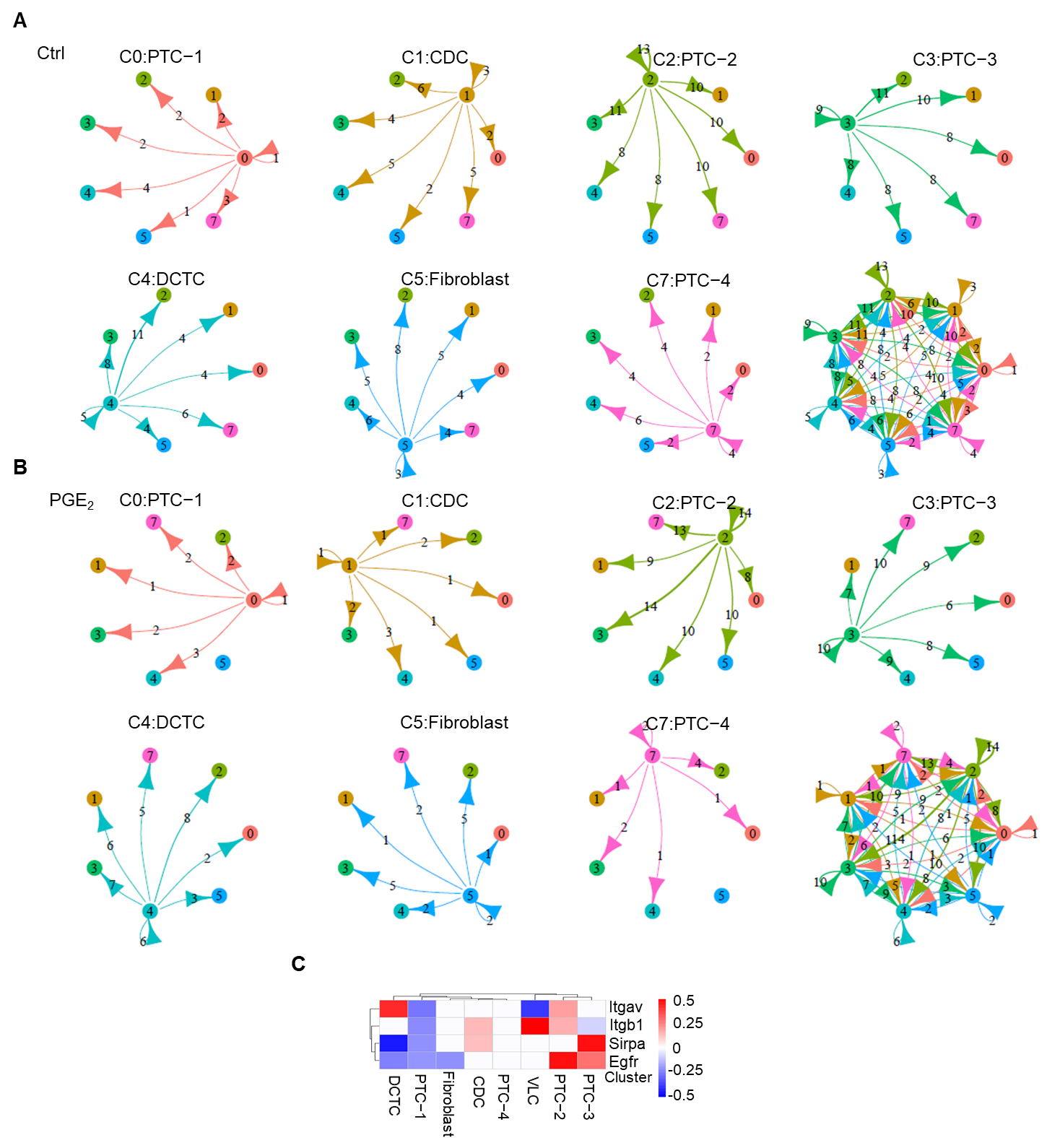


**Figure S7 Detailed view of growth factor-related ligands broadcast by each cell type.** (**A**) Detailed view of growth factor-related ligands broadcast by each cell type in the control group and those populations that express cognate receptors primed to receive a signal. The numbers indicate the quantity of ligand-receptor pairs for each inter-population link. (**B**) Detailed view of growth factor-related ligands broadcast by each cell type of PGE_2_ group and those populations expressing cognate receptors primed to receive a signal. Numbers indicate the number of ligand-receptor pairs for each inter-population link. The growth-related communication between PTC-2 and other cells were increased significantly with PGE_2_ treatment. (**C**) Heatmap showed the relative gene expression of DEGs enriched by functional blocks related to Vegfa receptors in each type, p value<0.05.


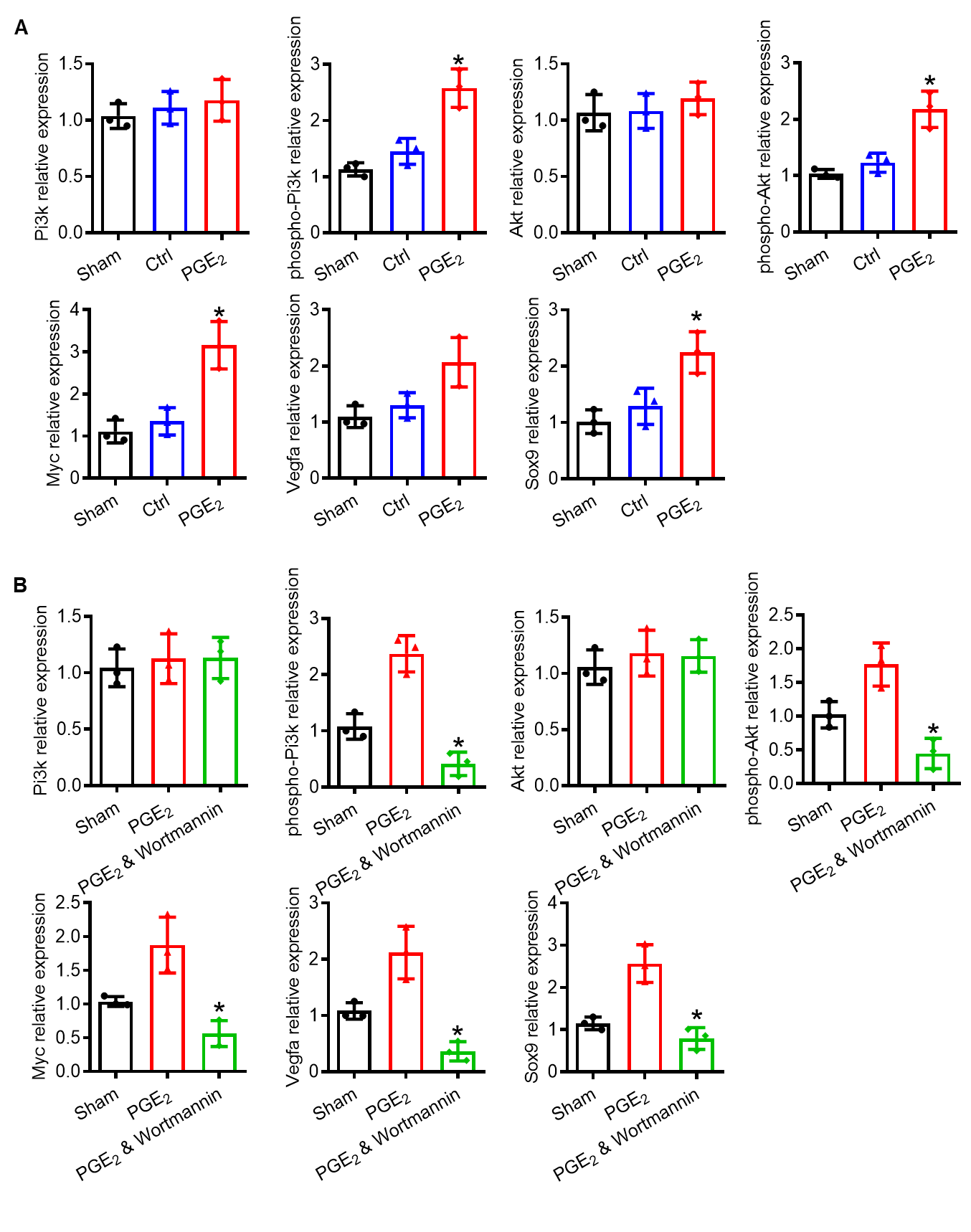


**Figure S8** (**A, B**) Quantification of the Western blotting analysis of Pi3k, p-Pi3k, Akt, p-Akt, Myc, and Vegfa expression in Sox9^+^ cells cultured on non-coated collagen and PGE_2_ matrix with/without the Pi3k-Akt inhibitor (wortmannin, 1 μM) for 48 h.

**
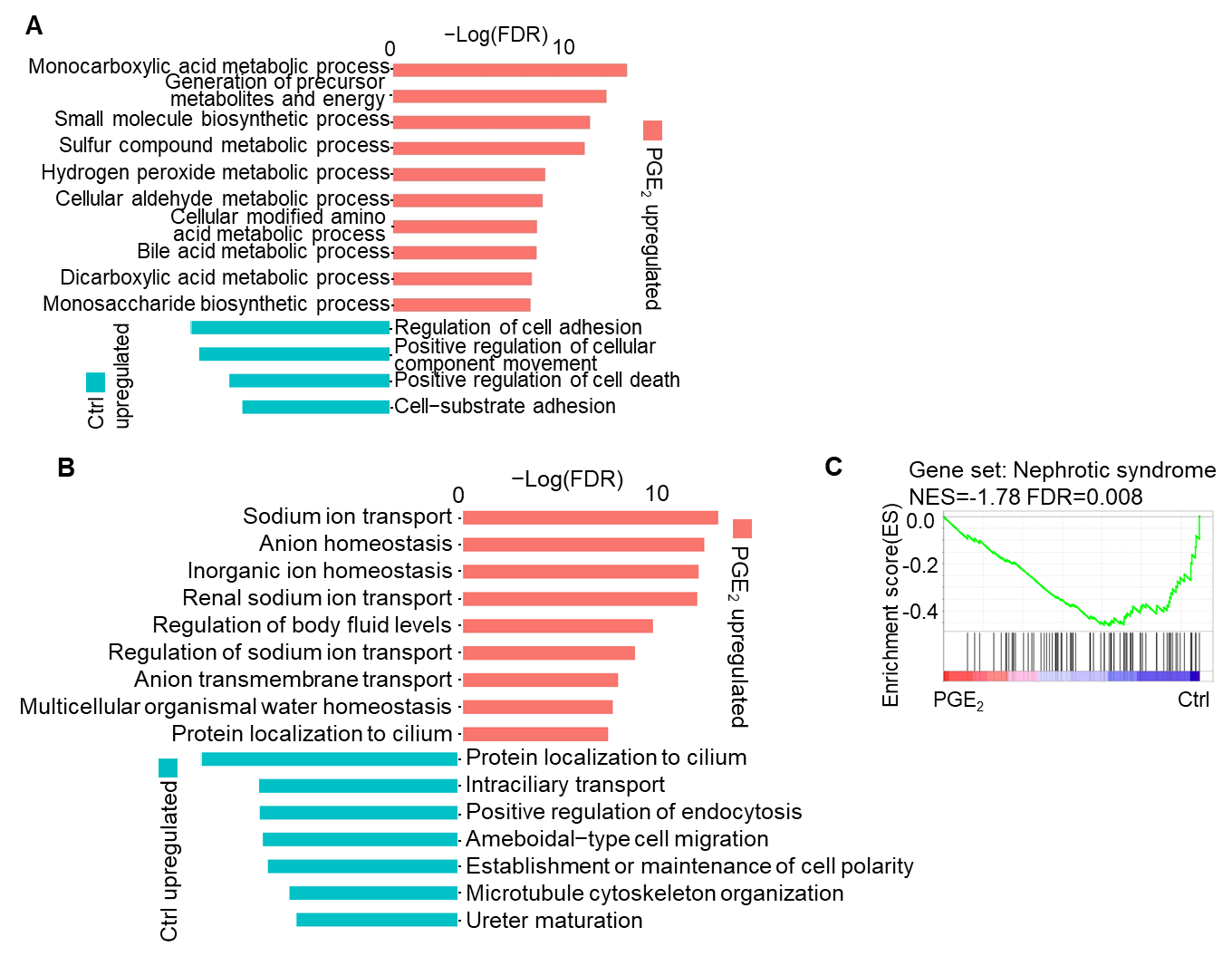
**

**Figure S9** (**A**) GO biological process enrichment analysis of DEGs identified in all PTCs (including PTC-1, PTC-2, PTC-3, and PTC-4). Metabolic homeostasis-related genes were mainly enriched in all PTCs treated with PGE_2_. log2 FC > 0.25, p value<0.05. (**B**) GO enrichment analysis of DEGs identified in the kidney phenotype gene panel comparing PGE_2_ with control groups. (**C**) GSEA enrichment plot of the gene dataset associated with nephrotic syndrome in PGE_2_-treated kidneys compared to control kidneys.

**Supplemental tables**

**Table S1** Statistics of differentially expressed genes among clusters in combined datasets of control and PGE_2_-treatment, related to Figure 2C. p_val: p value not adjusted for multiple test correction; avg_logFC: average log2-fold change. Positive values indicate that the gene is more abundant in the cluster; “pct.1” is the proportion of cells that express the gene in the target cluster, as labeled in the “cluster” column; “pct.2” is the proportion of cells that express the gene in the other clusters; p_val_adj: adjusted p value, based on Bonferroni correction using all genes in the dataset, used to determine significance; cluster: identity of cluster; gene: Ensembl gene ID

**Table S2** Statistics of differentially expressed genes between PTC-2 and other clusters in the combined kidney, related to Figure 3C and Figures 5A, B.

**Table S3** Pathway enrichment of genes upregulated between PTC-2 and other clusters in combined datasets, related to Figure 3D.

**Table S4** Statistics of differentially expressed genes among clusters in combined datasets, p value < 0.05, related to S6A-C.

**Table S5** Statistics of differentially expressed genes between PGE_2_-treatment and control kidney in each cluster, p value < 0.05, related to Figures 5 C, D, G and 6B.

**Table S6** Cross-talk of growth factor-mediated intercellular communication between cell types in control and PGE_2_-treatment, related to Figure 5H.

**Table S7** Pathway enrichment of genes upregulated between PGE_2_ and control in PTC-2, related to Figure 6A.

**Table S8** Cross-talk of cytokine-mediated intercellular communication among cell types in control and PGE_2_-treatment, related to Figure 8B.
